# Low and High Pressor Doses of Ang II Lead to Two Distinct Phenotypes of Hypertensive Heart Disease in Mice

**DOI:** 10.1111/apm.70132

**Published:** 2026-01-06

**Authors:** Diana Törmä, Tuisku Suoranta, Mimmi Rinta‐Harri, Jarkko Hytönen, Seppo Ylä‐Herttuala, Anna‐Kaisa Ruotsalainen

**Affiliations:** ^1^ A.I. Virtanen Institute University of Eastern Finland Kuopio Finland; ^2^ Heart Center and Gene Therapy Unit Kuopio University Hospital Kuopio Finland

**Keywords:** diastolic dysfunction, heart failure, hypertension, remodeling

## Abstract

Hypertension is a major contributor to cardiovascular diseases, being the most common comorbidity and the biggest risk factor in heart failure with preserved ejection fraction. Angiotensin II (Ang II) is a known hypertension and heart failure inducer in mice, but its role in the causality in phenotype development remains unclear. Here, hypertension was induced with low (LowA) or high (HighA) pressor doses of Ang II in mice. Both LowA and HighA groups demonstrated equal levels of hypertension with aortic dilatation and decreased aortic wall strain, but only HighA developed left ventricular hypertrophy with advanced cardiac dysfunction, demonstrating the hypertension‐independent effects of Ang II on myocardial remodeling. Alterations in electrical conductivity occurred similarly in both groups, with prominent ECG waveform aberrations. The study demonstrates two distinct hypertensive heart disease phenotypes induced by Ang II, providing a valuable preclinical framework that emphasizes the critical role of Ang II in diastolic dysfunction and vascular remodeling beyond its effects on the regulation of blood pressure.

## 1 Introduction

Cardiovascular diseases (CVD) are still the leading cause of morbidity, disability, and mortality worldwide [1], with hypertension being a dominant cause for their development [2]. The dysregulation of the renin‐angiotensin‐aldosterone system (RAAS) leads to elevated angiotensin II (Ang II) and aldosterone levels, promoting vasoconstriction and sodium retention, and thereby hypertension. Hypertension has detrimental short‐term and long‐term effects on the cardiovascular system, consequently promoting the development of cardiovascular and other end‐organ pathologies, such as chronic kidney disease [2, 3]. Chronic hypertension leads to blood vessel remodeling, elevating vascular resistance that increases the workload of the heart. The left ventricle (LV) compensates by enlargement of cardiomyocytes and undergoing concentric left ventricle hypertrophy (LVH) to maintain cardiac output (CO). Hypertension induces systemic and local oxidative stress and pro‐inflammatory activation in the myocardium, contributing to endothelial dysfunction that decreases nitric oxide production, impairing vasodilation and promoting vasoconstriction. This leads to coronary microvascular rarefaction, a reduction in the density of myocardial capillaries that promotes myocardial low‐grade ischemia and fibrosis, and subsequent arterial stiffness which reduces compliance, leading to impaired relaxation and elevated filling pressures during diastole [4].

Hypertension is the most prevailing comorbidity and the most common risk factor for the development of heart failure with preserved ejection fraction (HFpEF) [3, 5, 6]. HFpEF is a chronic condition with a poor prognosis marked by diastolic dysfunction, concentric LVH, and myocardial interstitial fibrosis [7]. Despite the established relationship of hypertension and HFpEF, the mechanisms of hypertension are challenging to study in humans, as the patients are usually diagnosed when the disease has already advanced and typically have comorbidities that aggravate HFpEF. Thus, the causality between hypertension and progression of cardiac remodeling and hypertrophy, and subsequent functional impairment remains unclear [8, 9]. Ang II inhibition has been widely and successfully used to treat hypertension in patients with both reduced and preserved ejection fraction (EF), but angiotensin‐converting enzyme inhibitors and Ang II receptor blockers have failed to reduce mortality in hypertensive HFpEF patients [4, 10].

Ang II has been widely used to induce hypertension, LV hypertrophy, and heart failure in mice [11, 12, 13] due to its long‐known Ang II receptor type 1 (AT_1_R)‐mediated vasoconstrictive, hypertrophic, pro‐inflammatory, and metabolic effects [14, 15]. Ang II mouse models have been utilized to model the disease in the absence of metabolic factors [16]. Although chronic Ang II infusion has been widely used in hypertension research [17], it is important to note that the level of hypertension [18] and cardiac remodeling have been reported to be dose‐dependent [19, 20]. Nevertheless, the effects of hypertension, AT_1_R expression, or Ang II dose on the diastolic function and HFpEF phenotype in mice are rarely described.

In this study, by using low and high pressor doses of Ang II, we demonstrate the development of two hypertensive phenotypes, characteristic features of hypertensive heart disease and HFpEF with or without concentric LVH. The study suggests that Ang II‐induced myocardial hypertrophy and diastolic dysfunction are not dependent on hypertension or AT_1_R expression. Additionally, this study provides a broad characterization of the diastolic dysfunction and myocardial and vascular remodeling in an Ang II‐induced mouse model, demonstrating the usefulness of this model to study the mechanisms and new treatment strategies for diastolic dysfunction and HFpEF.

## 2 Materials and Methods

### 2.1 Mice

C57Bl/6JOlaHsd 16–18‐week‐old mice (57 males) were obtained from Inotiv (Indianapolis, USA) and kept in a humidity‐ and temperature‐controlled environment with a 12 h light–dark cycle. Water and chow diet were provided *ad libitum*. Mice were group‐housed during acclimatization time of at least 1 week and single‐housed during the experiment. Hypertension was induced with low (0.5 mg/kg/day, further referred to as LowA) and high (1.5 mg/kg/day, further referred to as HighA) pressor doses of Ang II (1‐8, Asp‐Arg‐Val‐Tyr‐Ile‐His‐Pro‐Phe; #002‐12, Phoenix Pharmaceuticals Inc., CA, USA) for 4 weeks via Osmotic Minipumps (1004, DURECT Corporation, USA) implanted under isoflurane anesthesia subcutaneously into the mid‐scapular region according to the manufacturer's guidelines. Mice were provided with analgesics (Carprofen, 5 mg/kg) for postoperative pain relief. Animals were randomly assigned to disease and sham‐control groups. All experiments were approved by the National Animal Experiment Board of Regional State Administrative Agency of Finland and conducted according to ARRIVE guidelines.

### 2.2 Non‐Invasive Cardiovascular Physiology Assessment

Systolic and diastolic blood pressure measurements were acquired biweekly with CODA Non‐invasive Blood Pressure Monitor from the mouse tail (CODA Monitor, Kent Scientific, USA), with each animal individually acclimatized to the device prior to the study [21]. For acclimatization and stress reduction of the animals, each mouse was introduced to the translucent plastic tube prior to the study allowing unrestrained movement within the tube. After that, each mouse was separately acclimatized to the O‐cuff and VPR‐sensor, with a short measurement series conducted during two separate sessions. During the measurements mice were awake and kept loosely in the restraining tube on a warming pad at +37°C. After each measurement session, mouse was given a treat. Murine cardiac and aortic physiology assessment was done with transthoracic echocardiography (VEVO 3100 Ultrasound Imaging System, VisualSonics, Fujifilm Holdings, Japan) using MX400 transducer operating at 22–55 MHz. Mice were kept on a heating pad at +37°C under anesthesia (4.5% isoflurane induction, 1.5%–2.5% maintenance) in a supine position [22]. The heart was imaged at the mid‐ventricular level in B‐ and M‐modes in short‐axis view. For cardiac mass, corrected value was used, as is in clinics. In addition to automatically calculated values, relative wall thickness (RWT) was calculated as $\mathrm{RWT}=\frac{2\;x\;\mathrm{LV}\;\text{posterior wall in diastole}}{\mathrm{LV}\;\text{diameter in diastole}}$ [23]. Myocardial performance index (MPI) was automatically calculated as MPI = (IVCT + IVRT)/ET. Image for the assessment of diastolic function was obtained from the apical four‐chamber view with Tissue Doppler imaging at the septal annulus of the mitral valve. Trans‐mitral flow was acquired in Pulsed‐Wave (PW) Doppler mode. Aorta was imaged in modified parasternal long axis view in B‐mode. Analyses were carried out with VEVO Lab software 5.8.0. (VisualSonics, Fujifilm Holdings, Japan). Aortic (Ao) diameters were measured at the level of sinuses of Valsalva and from the proximal ascending aorta during the onset of ventricular depolarization (corresponding to PQ‐segment on ECG). Minimal and maximal aortic dimensions were measured during systole (at T‐wave) and during diastole (at R‐peak on ECG, respectively). Aortic compliance was evaluated from the proximal ascending aorta with the following parameters: $\mathrm{Ao}\;\text{strain}\;\left(\%\right)=\frac{\left(\mathrm{Ao}\;\text{diameter in systole}-\mathrm{Ao}\;\text{diameter in diastole}\right)\times 100}{\mathrm{Ao}\;\text{diameter in diastole}}$, Ao stiffness index = ln (Systolic pressure/Diastolic pressure)/Ao strain [24]. All echocardiography measurements were done from three consecutive breathing cycles. ECG at 1 kHz, corresponding to lead II, was obtained with VEVO 3100 Ultrasound Imaging System (VisualSonics, Fujifilm Holdings, Japan) during echocardiography imaging via limb electrodes on the mouse platform and analyzed with Kubios HRV Animal 3.4.3 ECG analysis program (Kubios Oy, Kuopio, Finland) from 30 s recordings as previously described [25]. Briefly, from the recordings, P‐ and T‐waves and QRSJ‐peaks were identified manually. The end of the T‐wave was determined at the place where it returns to its isoelectric point. PQ interval, QRS duration, QTc time width and R‐peak height were automatically calculated. All obtained ECG recordings were manually inspected for the identification of arrhythmias.

### 2.3 Blood Analysis

Blood was collected on Week 4 of the experiment. Blood chemistry analyses from heparin‐plasma were conducted by Movet Laboratory Services (Kuopio, Finland). For the fasting glucose, whole‐blood analysis was performed from 4 to 5 h fasted mice from the vena saphena with Ascensia ELITE (Bayer, Germany).

### 2.4 Histology and Immunohistochemistry

Mice were euthanized with CO_2_ and perfused with DPBS. For the histopathology assessment, heart, lungs, and kidneys were collected. Tissues were fixed in 4% paraformaldehyde overnight and transferred to 15% sucrose, embedded in paraffin blocks, and sectioned into 5 μm thin sections. All tissues were stained with HE‐stain. Additionally, cardiac tissues were stained with Podocalyxin (1:500; AF1556, goat anti‐mouse, R&D systems, USA), α‐smooth muscle actin (α‐SMA, 1:200, #19245, rabbit anti‐mouse, Cell Signaling Technology Inc. USA), and MAC‐3 (1:400; 553322; rat anti‐mouse, BD Pharminogen, USA) with hematoxylin background and Picrosirius red. Images of the whole mid‐LV (1/mouse) were obtained with Nikon DS‐Ri2 (Nikon, Japan) at 10× and 20× magnifications. Quantifications of the positively stained areas were performed from the whole mid‐LV for macrophages, endothelial, and fibrotic areas. Total endothelial area was quantified as podocalyxin positive area of the whole LV section. To determine regional differences in LV blood supply, inferolateral, inferior, septal, and anterior areas were imaged separately at magnification 20×, 4 images per section, and cross‐sectionally cut intramyocardial capillaries quantified. From the Picrosirius red stained mid‐LV sections, two biggest coronary arteries were identified and imaged at ×40 magnification. Perivascular fibrotic areas were quantified as positive area % of tunica intima‐media‐adventitia areas summed (luminal area was excluded from the measurements). Tunica intima‐media thickness was averaged from 15 measurements of the largest coronary artery per mouse. Cross‐sectionally cut cardiomyocytes and glomeruli were quantified from HE‐stained tissue sections. Areas of single cardiomyocytes from mid‐septal non‐fibrotic wall (10 measurements per ROI) were measured at magnification 40×. Glomerular and Bowman's capsule areas were quantified from at least 36 filtrating units per mouse at 10× magnification. LV fibrotic deposition and lung histology were also assessed visually. All images were analyzed with ImageJ FIJI [26].

### 2.5 qPCR Analysis

RNA extraction from mid‐LV cardiac and kidney tissues was performed for the qPCR analysis. Tissues were treated with TRI Reagent Solution (Invitrogen) and DNase treated with TURBO DNA‐free Kit (Invitrogen). RNA was reverse transcribed with RevertAid Reverse Transcriptase using Random Hexamer Primers (both Thermo Fisher Scientific). Expression levels of *Agtr1aI* (Mm.PT.58.41990121) for AT_1_R, *Atp2a2* (Mm.PT.58.5303089) for sarcoplasmic/endoplasmic reticulum Ca^2+^ ATPase (Serca2) and *Pln* (phospholamban, Mm.PT.58.43778023) were assessed as follows: qPCR ran on StepOnePlus (Applied Biosystems) using TaqMan Fast Advanced Master Mix (Applied Biosystems) with TaqMan chemistry‐based assays (Integrated DNA Technologies and Thermo Fisher Scientific). Relative gene expression was analyzed by using the 2^−∆∆Ct^ method using *Hprt1* (Mm.PT.39a.22214828) as a reference.

### 2.6 Statistical Analysis

Statistical analyses were performed with GraphPad Prism 10.0.2. For Ang II groups and control comparison, one‐way ANOVA with Dunnett's multiple comparisons post hoc test was performed. Changes within the same group at two different time points were analyzed with paired (unpaired when applicable) two‐tailed Student's *t* test. All values are presented as means ± SD. *p* < 0.05 was considered significant. All measurements, tests and analyses were performed blindly, with samples analyzed in random order on different days.

## 3 Results

### 3.1 Angiotensin II Induces Concentric Remodeling and Concentric Left Ventricular Hypertrophy in a Dose‐Dependent Manner but Independently of Hypertension

**FIGURE 1:**
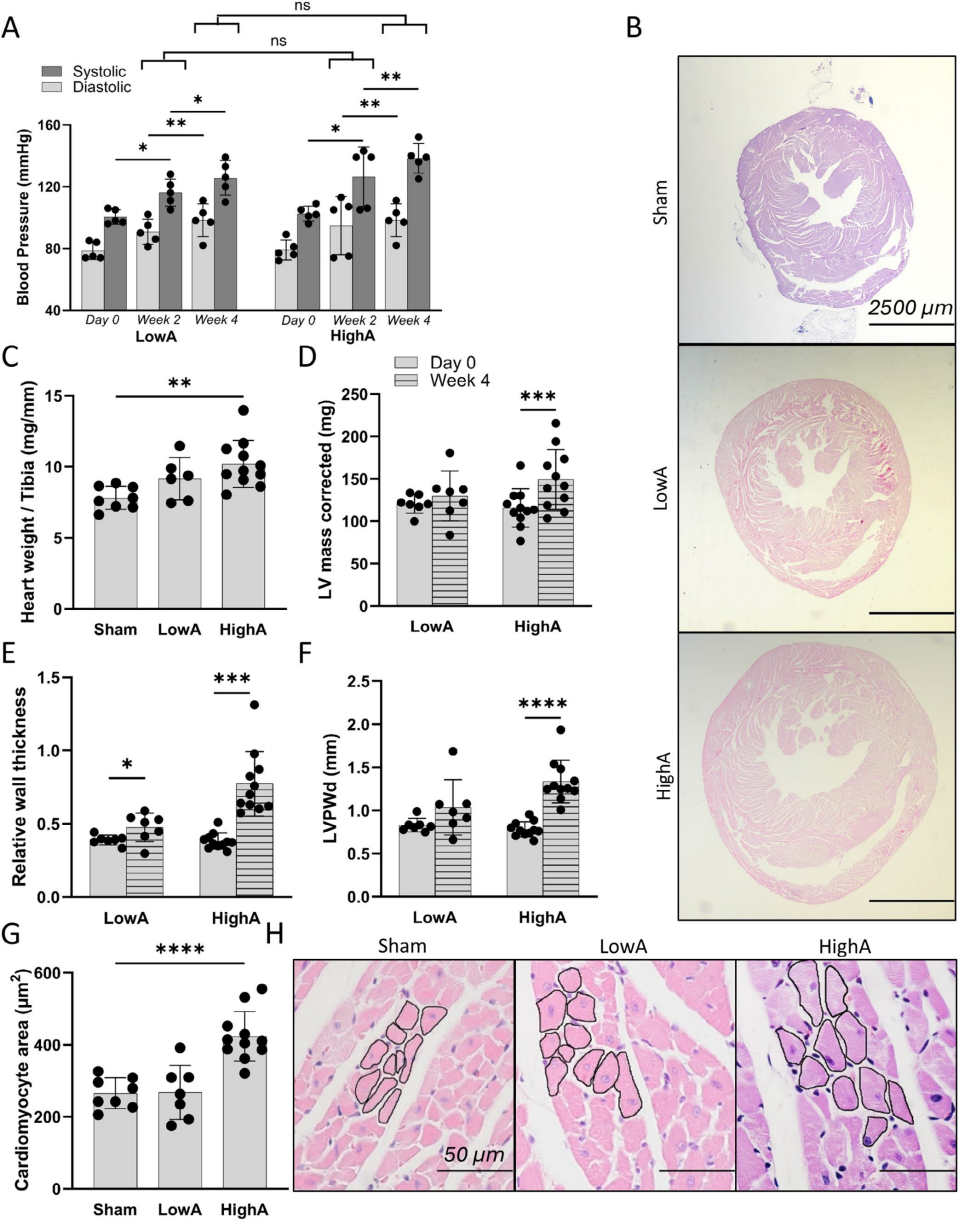
A dose‐dependent induction of myocardial concentric remodeling and hypertrophy, but not hypertension, by Ang II. (A) Gradual increase in both systolic and diastolic blood pressures measured with tail‐cuff method was achieved with s.c. 0.5 or 1.5 mg/kg/day angiotensin II infusions. (B) Representative cardiac LV cross‐sections stained with HE, scale bar 2500 μm. HighA group experienced an increase in (C) overall heart weight, (D) left ventricular mass (LV mass), (E) left ventricle posterior wall in diastole (LVPWd) and in (F) in relative wall thickness, but in LowA only relative wall thickness was increased. (G) Cardiomyocyte size measured from the myocardial septum showed cell hypertrophy in HighA group. (H) Representative images of septal cardiomyocyte areas. HE staining, scale bar 50 μm. Data presented as mean ± SD (*n* = 6–11 mice/group; for blood pressure measurements *n* = 5 for all groups; Statistical analysis was performed by One‐way ANOVA with Dunnett's post hoc test (A, C, G) and paired two‐tailed Student's *t* test (D, E, F); for A also unpaired two‐tailed Student's *t* test was performed for comparison of two dose groups on same the timepoint; *p < 0.05, **p < 0.01; ***p < 0.001; ****p < 0.0001; ns, non‐significant).

LowA and HighA pressor doses of Ang II were used to mimic gradual progression of hypertensive heart disease in mice. Blood pressure was measured biweekly, and both systolic and diastolic pressures were increasing gradually during 4 weeks throughout the experiment (Figure 1A, S1). Interestingly, blood pressure was independent of Ang II pressor dose as it rose equally in both LowA and HighA angiotensin groups.

To assess the potential systemic and metabolic effects of Ang II, fasting plasma glucose and liver enzyme levels were measured after 4 weeks of Ang II infusion, but no difference between the study groups was detected (Figure S2A,B). Body weight decrease occurred only in HighA on week four of infusion (Table S1).

Notable change in LV and whole heart size can be observed from the representative pictures of HE‐stained cross‐sections of myocardium (Figure 1B) demonstrating the gradual advancement of concentric hypertrophy by both LowA and HighA. Heart weight in relation to tibia length (Figure 1C and Table S2) and LV mass (Figure 1D) were increased in HighA, but not significantly in LowA. In addition, RWT was significantly increased in both LowA and HighA (Figure 1E), but only HighA had increased LV posterior wall thickness in diastole (Figure 1F). Finally, HighA induced cardiomyocyte hypertrophy as the size of the cardiomyocytes was increased in the HighA group in comparison to sham and LowA groups (Figure 1G,H). Additional systolic and diastolic parameters are presented in Table S3.

### 3.2 Cardiac Remodeling and Fibrosis Alter Electrical Impulse Propagation in the Affected Myocardium

**FIGURE 2:**
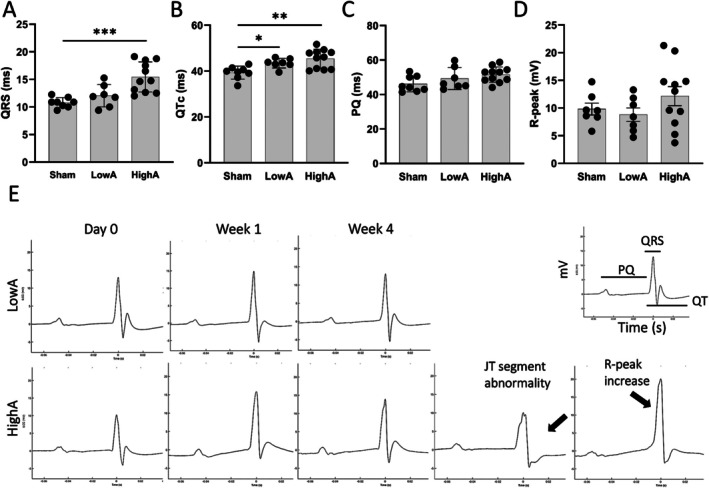
Pathological alterations in electrical conductivity of the heart in both hypertensive groups. (A) Ventricular depolarization (QRS). (B) Total ventricular depolarization and repolarization time corrected (QTc). (C) Atrial depolarization (PQ). (D) R‐wave peak. (E) ECG‐waveform transformation in LowA and HighA during the experiment. Data presented as mean ± SD (*n* = 7–11 mice/group; Statistical analysis was performed by One‐way ANOVA with Dunnett's post hoc test; *p < 0.05, **p < 0.01; ***p < 0.001).

ECG analysis showed significant prolongation in ventricular depolarization time (QRS) in HighA (Figure 2A). However, total ventricular depolarization and repolarization time, QTc, was significantly prolonged in both hypertensive groups (Figure 2B). Atrial depolarization (PQ) did not show any difference between the dose groups and controls (Figure 2C). An elevation in the R wave peak was noticed in 3/11 mice from HighA, but the group mean was not statistically significant (Figure 2D). From all ECG recordings, only one case of arrhythmia was detected in the HighA group. No sudden deaths occurred during the experiment. Representative ECG recordings from both hypertensive groups (Figure 2E) demonstrated a notable transformation of the ECG occurring during Week 1, with J wave, a characteristic feature of the murine ECG, depressing or disappearing, and/or declining JT‐segment. Such changes were seen in 4/8 of mice in LowA and 8/11 in HighA. Interestingly, recovery of the J‐wave and/or JT‐segment were visible already during Week 2, occurring in all mice in the LowA group. Mice with abnormal J‐waves in HighA did not show such a recovery, with changes of the JT‐segment in these animals persisting until the end of the experiment.

### 3.3 Impairment of the Ventricular Filling and Reduction in Ventricular Compliance Demonstrate Progression Towards Diastolic Dysfunction in Ang II Dose‐Dependent Manner

**FIGURE 3:**
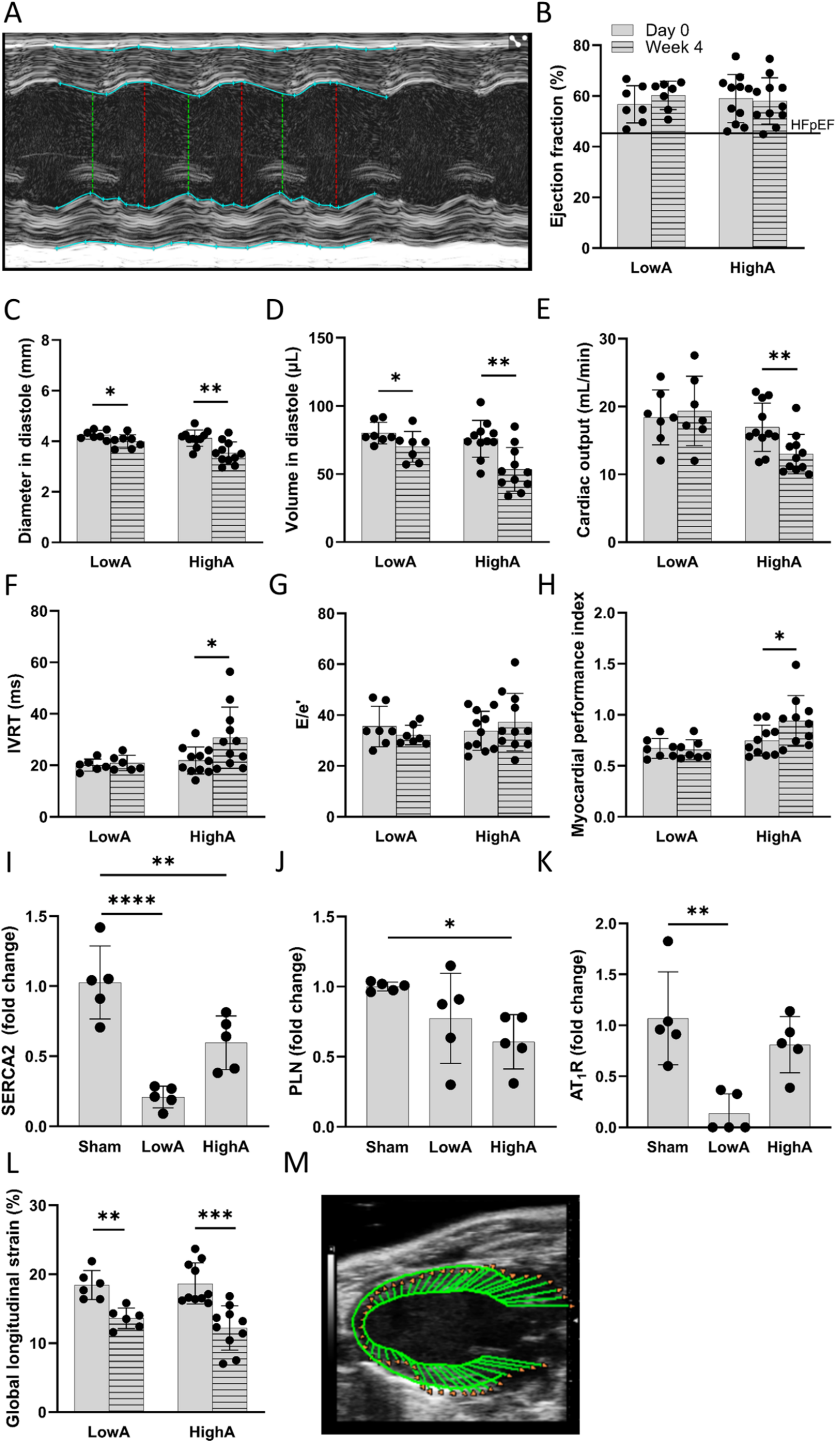
Cardiac physiology assessment shows angiotensin II dose‐dependent and—independent differences in hypertensive phenotypes. (A) M‐mode of echocardiography imaging with LV measurements. (B–D) Summary of echocardiography data showing normal ejection fraction remaining within HFpEF limits throughout the study. LV filling impairment occurred in both hypertensive groups as diastolic diameters and volumes dropped significantly. (E) HighA also showed signs of a failing heart in significant drop of cardiac output. Signs of diastolic (F, G) and systolic (H) dysfunction in angiotensin groups (*n* = 7 in LowA; *n* = 11 in HighA). (I, J) QPCR analysis showed the expression of the key regulators of cardiac contractility, SERCA2 and PLN, contributing to myocardial stiffening and poor relaxation in LowA and HighA groups (*n* = 5 for all groups). (K) AT_1_R expression in the hearts. (L) Global longitudinal strain analysis from echocardiography data showing significant deformation in the LV in both dose groups. (M) Representative image of Day 0 strain analysis from the LV long‐axis view. Data presented as mean ± SD (Statistical analysis was performed by paired two‐tailed Student's *t* test (A–G) and One‐way ANOVA with Dunnett's post hoc test (H–J); *p < 0.05, **p < 0.01, ***p < 0.001, ****p < 0.0001). AT_1_R, Angiotensin II type 1a receptor; E/e', early mitral inflow velocity to mitral annular early diastolic velocity ratio; HFpEF, heart failure with preserved ejection fraction; IVRT, isovolumic relaxation time; LV, left ventricle; PLN, phospholamban; SERCA2, sarcoplasmic/endoplasmic reticulum Ca^2+^‐ATPase.

Echocardiography was performed to assess the diastolic and global cardiac functions before and after 4 weeks of Ang II infusion (Figure 3A). Neither low nor high dose affected EF, which remained similar (60.2% ± 5.2% in LowA, 58.0% ± 8.7% in HighA) in all groups (Figure 3B). Significant drop in diastolic diameter and volume occurred in both hypertensive groups indicating the impairment of ventricular filling (Figure 3C,D). Decrease in CO in HighA, but not in LowA was observed despite stable EF (Figure 3E). Also, prolonged myocardial relaxation was noticed in HighA, with a significant prolongation of isovolumic relaxation time (IVRT) (Figure 3F). Interestingly, early mitral inflow velocity to mitral annular early diastolic velocity ratio (E/e') showed group variability, being increased in 1/7 mice in LowA and 7/11 mice in HighA, demonstrating a gradual shift towards impaired LV relaxation and diastolic dysfunction (Figure 3G). Assessment of the global cardiac function with myocardial performance index showed an impairment only in HighA group (Figure 3H). To further assess the LV contractility, the gene expression of *Atp2a2* (Serca2) (Figure 3I) and *Pln* (Figure 3J) were measured on mRNA level, and the significant decline in the expression of both genes was noticed in HighA. In LowA only Serca2 was reduced, but *Pln* expression was equal to sham mice. Also, the expression of *AT*_*1*_ *R* (Figure 3K) was measured from the heart, and in the LowA group the expression was significantly reduced. HighA group showed expression level equal with the sham mice. Global longitudinal strain analysis showed significant decline in both Ang II dose groups (Figure 3L,M).

### 3.4 Myocardial Macrophage Activation Is an Early Response to Hypertension, but Myocardial α‐ Smooth Muscle Actin Activation and Fibrosis Are Detected in Hypertrophic Myocardium Only

**FIGURE 4:**
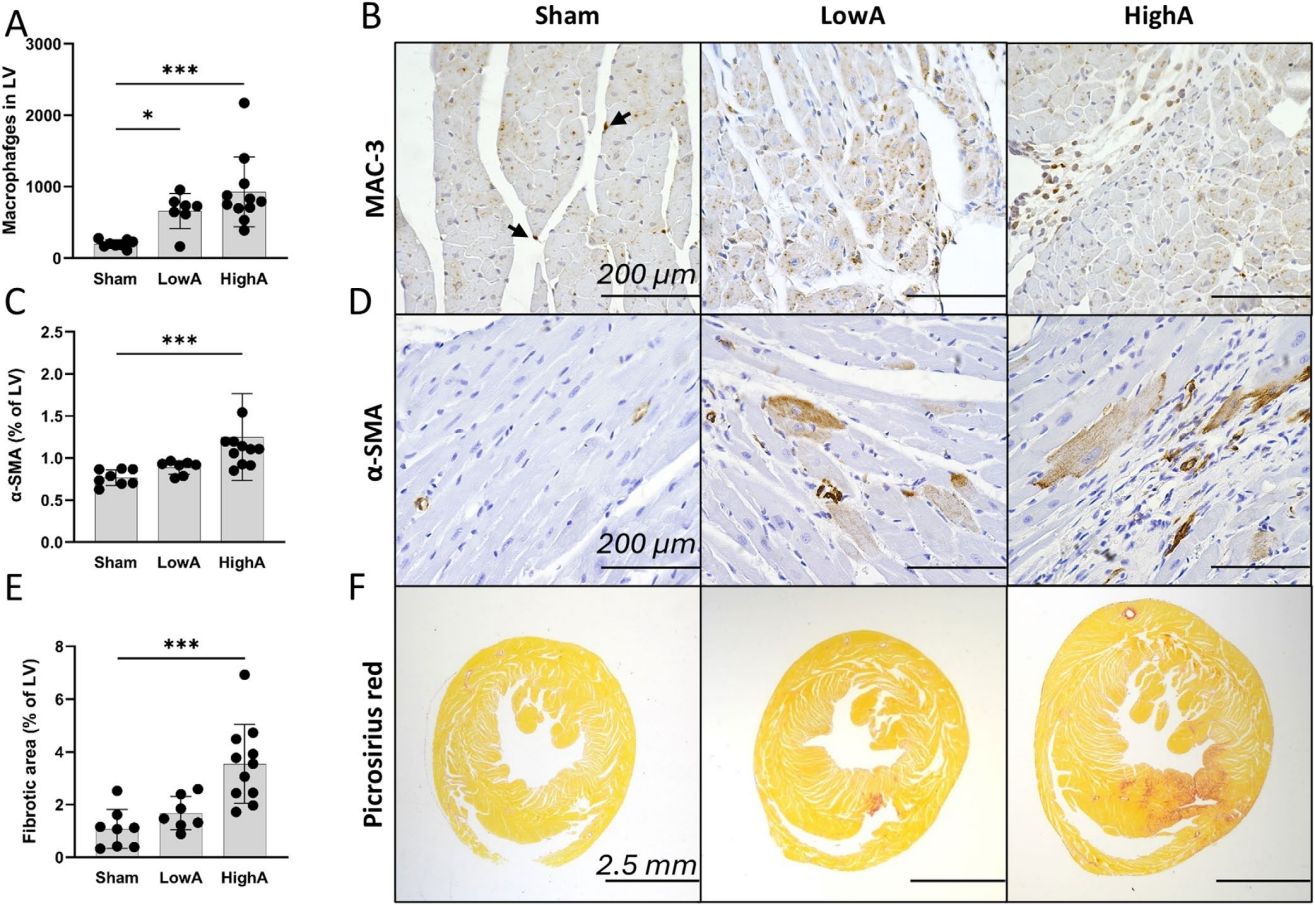
Hypertension leads to myocardial inflammatory activation, but myocardial α‐ smooth muscle actin activation and fibrosis are detected in hypertrophic myocardium only. (A, B) Hypertension caused increase in number of MAC‐3 positive macrophages in mid‐LV by Ang II. Notice elongated shape and position of single resident‐macrophages (arrow) in sham versus rounded ones gathered within fibrotic areas of hypertensive groups (scale bar 200 μm). (C, D) Rise in α‐SMA expression and growing presence of positively stained cardiomyocytes indicate hypertensive response in the affected myocardium (scale bar 200 μm). (E, F) Significant increase in myocardial fibrosis in HighA but not LowA (Picrosirius red staining, scale bar 2.5 mm). Data presented as mean ± SD (*n* = 7–11 mice/group; Statistical analysis was performed by One‐way ANOVA with Dunnett's post hoc test; *p < 0.01, ***p < 0.001).

As hypertension led to myocardial stiffening and impaired cardiac function in both LowA and HighA groups, we next analyzed the histopathology of the myocardium. Myocardial macrophage number, detected as MAC‐3 positive cells, was increased in both dose groups in comparison to sham (Figure 4A). In addition, the macrophage morphology (Figure 4B) was modified by hypertension, with resident macrophages in the sham group being elongated and residing within the intact myocardium, whereas migrated macrophages in the hypertensive mice were round‐shaped and found in the vicinity of the fibrotic areas. Increased α‐SMA positive area in the myocardium (Figure 4C,D) was seen in HighA when compared to LowA and sham mice. Also, an elevated interstitial cardiac collagen content was detected in HighA in comparison to sham and LowA groups (Figure 4E) and a mild increase in collagen deposition in LowA was also found. In both groups, the collagen deposition was detected in the septum and also in the papillary muscles and around the larger coronary arteries (Figure 4F and Table 1).

**TABLE 1:** The analysis and scoring of collagen depositions in LV by visual assessment from Picrosirius red stained mid‐LV sections.

Collagen deposition in LV	Sham	LowA	HighA
*Inferoseptal*	−	+	+
*Anteroseptal*	−	+/−	+/−
*Inferolateral*	−	+/−	+/−
*Perivascular*	−	+/−	+

*Note:* “+” present in all, “−” absent in all, “+/−” absent in some.

### 3.5 Impaired Macrovascular and Microvascular Structure and Function Contribute to Diastolic Dysfunction in the Presence and Absence of Left Ventricular Hypertrophy

**FIGURE 5:**
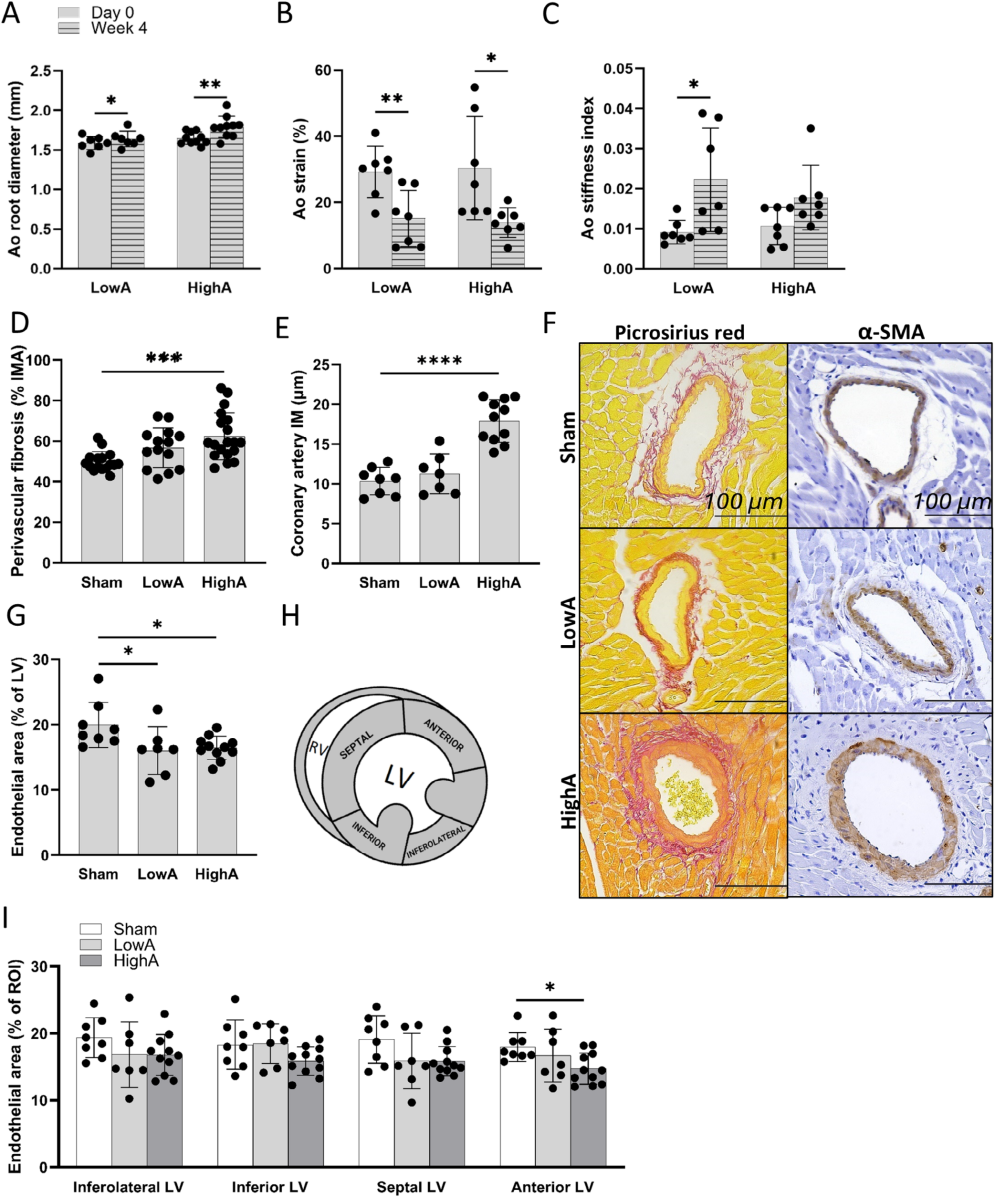
Impaired macrovascular and microvascular structure and function contribute to diastolic dysfunction in the presence and absence of left ventricular hypertrophy. (A) Aortic root diameter was imaged with echocardiography and measured from the modified parasternal long axis view in B‐mode from the proximal ascending aorta during the onset of ventricular depolarization. (B, C) Changes in both aortic strain and stiffness index demonstrate loss of aortic wall elasticity in LowA. (D) Accumulation of perivascular collagen. (E) Coronary artery intima‐media (IM) thickening. (F) Representative images of coronary arteries stained with Picrosirius red and α‐SMA show ongoing remodeling of vascular walls (scale bars on all images 100 μm). (G) Podocalyxin‐positive endothelial area was measured demonstrating overall decrease in mid‐LV capillary number. (H, I) Also, regional differences in capillary areas were noticed within the myocardium. Data presented as mean ± SD (*n* = 7–11 mice/group; Statistical analysis was performed by paired two‐tailed Student's *t* test (A–C) and One‐way ANOVA with Dunnett's post hoc test (D, E, G, I); *p < 0.05, **p < 0.01, ***p < 0.001, ****p < 0.0001). Ao, aorta; IM, intima‐media; IMA, intima‐media‐adventitia.

Hypertension was similar in both LowA and HighA groups, but a more advanced diastolic dysfunction with myocardial hypertrophy and fibrosis was detected in the HighA group. Next, we characterized macrovascular and microvascular morphology and function in the study groups. Firstly, hypertension led to a dilatation of the aortic root as much as 3.1% ± 2.2% and 8.8% ± 6.2% in LowA and HighA groups, respectively (Figure 5A). The elastic properties of the aortic wall were impaired, as aortic wall movement was compromised in both LowA and HighA groups with a significant drop in aortic strain (Figure 5B). A significant rise in aortic stiffness index in the LowA group and a minor elevation in the HighA group (Figure 5C) also supported the data demonstrating the dependency of arterial elasticity on the developing chronic hypertension and progression of the diastolic dysfunction. Coronary artery remodeling in both the tunica intima‐media layers as well as in the perivascular area was detected with hypertension in an Ang II dose‐dependent manner. Periarterial fibrosis (Figure 5D) and coronary artery medial thickness (Figure 5E) were both significantly increased in HighA. Reorganization of medial smooth muscle cells was also present, as seen in the representative pictures (Figure 5F) of LowA and HighA groups. Finally, the effect of hypertension on microvasculature was analyzed, and we found that LV capillaries also responded to hypertensive stress with a 19.7% ± 16.9% loss in capillary area in LowA and a similar 17.5% ± 8.2% drop in the HighA group (Figure 5G). A closer look at capillary areas within LV segments (Figure 5H,I) revealed regional differences in myocardial blood supply, with more alterations occurring in the septal and anterior segments.

### 3.6 Renal Glomerular Enlargement Is Dependent on Ang II Dose, but Not Renal AT_1_R Expression or Systemic Hypertension

**FIGURE 6:**
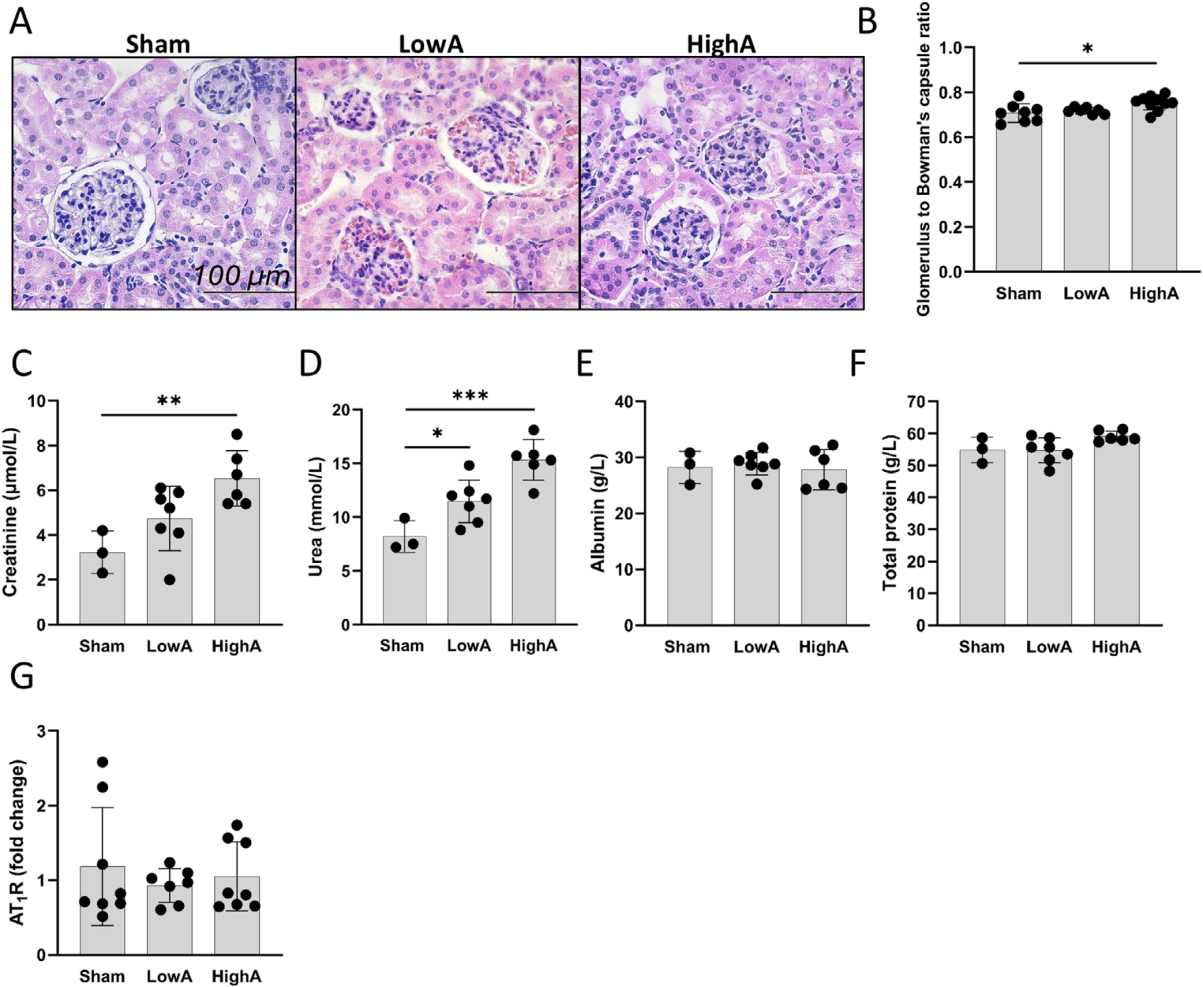
Renal glomerular enlargement is dependent on Ang II dose, but not renal AT_1_R expression or systemic hypertension. (A) Representative HE staining of Bowman's capsules. (B) Dose‐dependent increase seen in glomerulus to Bowman's capsule ratio (scale bar 100 μm). (C, D) Kidney filtration capacity decline was seen in increased levels of plasma creatinine and urea. (E, F) No change in albumin or total protein levels was detected. (G) QPCR showed no difference in AT_1_R expression between hypertensive groups and controls. Data presented as mean ± SD (*n* = 7–11 mice/group; Statistical analysis was performed by One‐way ANOVA with Dunnett's post hoc test; for glomeruli pathology, 36–68 filtrating units were measured and averaged; *p < 0.05, **p < 0.01, ***p < 0.001). AT_1_R, Angiotensin II type 1a receptor.

To investigate systemic effects of hypertensive heart disease and cardiac diastolic dysfunction we examined kidney and lung histopathology. Kidneys demonstrated a significant increase in glomerulus to Bowman's space ratio (Figure 6A,B) in an Ang II dose‐dependent manner. To further assess the kidney filtration capacity, we analyzed plasma creatinine, urea, albumin and total protein amounts. Creatinine (Figure 6C) and urea (Figure 6D) were both elevated in HighA, and in LowA only urea was significantly increased. Plasma albumin (Figure 6E) and total protein amount (Figure 6E) were not affected by Ang II showing that kidney filtration capacity is still proficient for larger molecules. The expression of AT_1_R in kidney was also measured, but the expression remained similar within the groups (Figure 6G). Finally, lung histopathology (Figure S3) showed alveolar septal thickening of various degrees in both Ang II dose groups, which was seen in 4/7 mice in LowA and in all mice in HighA group.

## 4 Discussion

The mechanisms and interplay of hypertension with myocardial and vascular remodeling and its progression towards diastolic dysfunction and HFpEF still require clarification. In this study, we demonstrated two distinct phenotypes of hypertensive heart disease in mice induced by low and high pressor doses of Ang II. Here, both Ang II doses produced comparable hypertension but distinct cardiac and vascular remodeling. Interestingly, the induction of hypertension was not fully Ang II dose‐dependent, as both groups experienced comparable levels of hypertension, likely leading to similar loss of aortic compliance and a decrease in myocardial capillaries in both groups. Conversely, the high‐dose group exhibited hallmarks of early HFpEF, such as advanced coronary artery remodeling, significant myocardial hypertrophy and stiffening, and a progressive impairment in diastolic function. Thus, prominent myocardial hypertrophy and diastolic dysfunction showed a dependency on Ang II dose, but not hypertension, suggesting that Ang II is mediating LV fibrosis and cardiomyocyte hypertrophy via its other mechanisms such as the regulation of inflammation and cell proliferation. Moreover, we demonstrated that systemic effects with renal impairment and alveolar septal thickening in lungs can be induced in an Ang II dose‐dependent manner. Overall, Ang II infusion is an effective method for inducing hypertensive heart disease with myocardial hypertrophy and diastolic dysfunction in mice, resembling the clinical HFpEF phenotype [12, 13]. Importantly, the induction of hypertrophy, diastolic dysfunction, and kidney pathologies are not dependent on hypertension only, or the expression of AT1R in the myocardium or kidney, respectively. The study suggests that while hypertension is known to play a critical role in vascular remodeling and impairment, and in the development of hypertensive heart disease, contributing to HFpEF development, myocardial hypertrophy and Ang II‐mediated mechanisms are prominent mediators of disease progression towards diastolic dysfunction and HFpEF in mice.

Ang II has several prominent effects on the cardiovascular system due to its ability to enhance vasoconstriction, hypertrophy, fibrosis, and inflammation [27]. Its diverse effects are mediated through AT1R, a G‐protein coupled receptor expressed in many tissues within and outside the cardiovascular system, activating multiple intracellular transduction cascades through Ras/Raf/MEK/ERK, PI3K/Akt‐PKB/mTOR, and Rho/ROCK pathways, with opposing effects mediated by AT2R [28]. In the present study, Ang II induced gradual hypertension over the duration of 4 weeks, but surprisingly, induction attained comparable blood pressure levels within this pressor dose range, as seen in both dose groups. It has been previously reported that very low doses of Ang II, such as 0.15 mg/kg/day, do not elevate blood pressure and cause only minimal cardiomyocyte hypertrophy [20]. Similarly, high doses of Ang II (2.4 mg/kg/h) are unlikely to promote hypertension but, in addition to causing myocardial hypertrophy, induce myocardial injury with massive myocardial necrosis and fibrosis [29]. In the present study, with the higher Ang II dose, we also detected mostly periarterial or septal LV fibrotic depositions—a finding that seemed to be independent of hypertension but dependent on the Ang II dose. However, the infiltration of macrophages, the modifications of coronary arteries and capillaries, aortic root dilatation, the reduction in aortic strain, and the increase in aortic wall stiffness index were detected equally in both LowA and HighA groups indicating dependence on hypertension rather than on the Ang II dose. Notably, both Ang II and hypertension have similar primary effects, such as the activation of oxidative stress, which further promotes pro‐inflammatory and pro‐fibrotic activation in the vasculature. Ang II has also been reported to directly affect cell proliferation and growth, such as the proliferation of vascular smooth muscle cells in vivo [30]. This could also explain the vascular remodeling, such as thickening of the coronary medial layer in an Ang II dose‐dependent manner, observed in the present study. Overall, our Ang II model closely mimics the hypertensive heart disease phenotype and the progression towards the clinical HFpEF phenotype independently of hypertension, which is potentially mediated via other effects of Ang II.

Interestingly, in our study the induction of cardiac hypertrophy and myocardial stiffness seemed to be reliant on Ang II dose rather than hypertension. This suggests that Ang II dependent effects on the myocardium are possibly mediated via secondary activation of various kinases and growth factors, which is also in line with other studies [27, 28]. Ang II doses ≥ 0.864 mg/kg/day appeared to cause an increase in LV size [31, 32, 33, 34, 35]—a finding similar to our results. A previous study by Regan et al. [36] showed that the HFpEF phenotype with impaired relaxation didn't depend on hypertension or LV hypertrophy with 0.2 mg/kg/day of Ang II. Although LVH is often assumed to be the hallmark feature of murine HFpEF, clinical studies have demonstrated that less than half of the HFpEF patients have LVH despite hypertension [37, 38] indicating the existence of alternative routes for LVH pathogenesis [39] and other roots to functional LV impairment and diversity of clinical phenotypes. A previous study has reported myocardial stiffness to be unaltered in hypertensive patients without HFpEF, whereas in hypertensive HFpEF stiffness was titin‐ and collagen‐dependent. Interestingly, the same study showed significant RWT and LV mass increases in hypertensive patients with and without HFpEF [40]. α‐SMA positive cardiomyocytes were previously noticed in rat cardiomyocytes in vitro [41], and in vivo hypertrophic rat hearts as a cellular response to hemodynamic overload [42], although Suurmeijer et al. [43] did not have similar findings in humans. We observed α‐SMA positive cardiomyocytes in both Ang II dose groups, suggesting that pathological cytoskeletal alterations in cardiomyocytes might be an initial manifestation of myocardial remodeling as cardiomyocytes become larger, prior to other visible structural changes within myocardium, such as periarterial or myocardial interstitial fibrosis or microvascular alterations, as was seen in LowA group. Such early cardiomyocyte modifications might be exclusive to rodents.

It has been suggested that early diastolic dysfunction may develop prior to LVH in both humans and mice [44]. Our study demonstrated impaired LV filling in LowA in the absence of LV hypertrophy, but with early concentric remodeling. In HighA, however, progressed myocardial remodeling with significant LV hypertrophy aggravated the progression of diastolic dysfunction, as we saw impaired myocardial relaxation (IVRT) and impaired LV filling, although no significant change in LV filling pressure (E/e') was observed. Thus, it seems that hypertrophy has a critical role in the development of diastolic dysfunction in mice, and the induction is mediated via Ang II‐dependent effects potentially on cardiomyocyte TGF‐signaling and mitochondrial function. Hypertension clearly plays a role in the pathogenesis, but it does not have a clear effect on myocardial hypertrophy.

Our results showed EF of 45%–50% in five control mice, two mice in LowA and three mice in HighA group on Day 0. Considering the anesthesia applied, heart rate of the mice [22], measurement variability [45] and intraoperator variability of the ultrasound results, we determined EF of > 45% to represent a baseline value, while in humans normal value for EF is considered to be ≥ 50%. In addition to EF, E/e' ratio is an important additional diagnostic parameter of clinical HFpEF [45]. In our study, no changes were detected between the groups in E/e'. Increase in E/e' ratio appears to be a non‐compulsory feature in HFpEF patients, as recent study by Shah et al. [37] showed that rise in E/e' was only present in 53% of patients with EF > 45%. Our study shows that even in HighA, E/e' increase was observed only in some mice, indicating the exacerbation of diastolic dysfunction and progression towards a clear HFpEF phenotype. Early to late diastolic transmitral flow velocity (E/A) for the determination of diastolic dysfunction has been used in clinical work but was not measured in the present study due to unreliability of the measurement in mice [22, 46, 47].

To further analyze the myocardial contractility, we found a decrease in *Serca2* expression in LV of both dose groups despite differing cardiac hypertrophy and fibrosis levels. In addition, the expression of *Pln* was also reduced in the HighA group presenting more severe cardiac phenotype. Alterations in PLN‐SERCA2 interaction and expression, seen in heart failure patients, cause impairment in cardiac relaxation and contraction [48]. Our results were supported by the observation of reduced global longitudinal strain, indicating a decline in cardiac contractility and abnormal myocardial wall dynamics. Interestingly, pathological changes in global longitudinal strain are not only present in heart failure patients [45], but have also been observed in patients with hypertension [49]. Changes in myocardial function, therefore, seem to occur early on in the disease progression, prior to other observable myocardial features, such as increase in cardiac mass, interstitial fibrosis or diastolic dysfunction, as was seen in our study in the LowA group.

Dilatation of the proximal aorta is seen in approximately 10% of hypertensive patients [50]. Aortic parameters are not considered a diagnostic criterion for clinical HFpEF, although pathological alterations in aortic wall dynamics have been found in patients with asymptomatic diastolic dysfunction [51] and early‐stage HFpEF [52], indicating that aortic pathology develops prior to HFpEF as hypertension persists. This also suggests that the evaluation of aortic stiffness may be a possible marker of the initial, subclinical HFpEF manifestation. Our results demonstrate that similar aortic modifications occur within the given Ang II dose range concomitantly with progression of hypertrophy and diastolic dysfunction in both hypertensive groups, indicating that this mouse model could be further utilized to investigate the related molecular mechanisms and therapeutic approaches for hypertension, diastolic dysfunction, and early HFpEF interventions.

Evaluation of the ECG is an important tool in a clinical setting for the assessment of cardiac function and one of the initial steps in HF diagnosis [45]. Abnormal baseline ECGs predict poor outcomes in patients with HFpEF [53]. Excess fibrotic accumulation within the LV—associated with hypertensive heart disease, HFpEF, and aortic stiffness in humans [54]—creates disturbances in impulse propagation routes [55]. Publications on murine ECG in CVDs, however, are scarce, making the comparison challenging. Contrary to our findings, Jacobsen et al. [56] noticed no difference in the electrophysiological profile in hypertensive HFpEF mice. However, this might be due to the younger age of the mice and a different approach in HFpEF induction. Mice with myocardial remodeling caused by a higher dose of Ang II showed QRS and QT prolongation in ECG [57], similar to our findings. Interestingly, in young mice, the QT interval showed sex dependency at the baseline [58]. A study by Merentie et al. [25] showed JT/T‐segment changes in ECG in mice with acute myocardial infarction. Murine ECG assessment, especially changes in ECG morphology in hypertensive mice with diastolic dysfunction, is rarely provided. Our findings demonstrated prolonged ventricular conduction, as is seen in clinical presentation of HF [45]. However, only a single case of arrhythmia was observed in this study. We also noticed alterations in the JT/T‐segment, correlating with the given Ang II dose and the level of hypertrophy and fibrosis. The changes in ECG waveforms of HighA but not LowA animals might thus be explained by greater LV hypertrophy and remodeling. Thus, in our study, the phenotype of the LowA group represents hypertensive heart disease with initial structural cardiac and aortic modifications and with progressing diastolic dysfunction, whereas HighA represents more advanced HFpEF with systemic effects.

Our data shows that Ang II‐induced chronic hypertension has systemic effects, greatly altering renal structure and function. It is known that kidney dysfunction contributes to HFpEF progression, and patients with chronic kidney disease demonstrate graver HFpEF phenotype [3]. Pathological development in both appears to be bidirectional [3], and our results also show progressing renal impairment in both Low and High Ang II‐groups as a direct impact of changes in hemodynamic and micro‐ and macrovasculature.

### 4.1 Limitations of the Study

There are several limitations to this study. Firstly, this study used only adult male mice, limiting range in sex and age diversity. It is known that HFpEF is more prevalent in females than in males in humans [3], however, with hypertension it is exactly the opposite [59], with similar findings in rodents [60]. A recent study by Walsh‐Wilkinson et al. [61] showed mouse sex‐ and age‐related differences in LV regional strain in the presence of Ang II‐induced hypertrophy, demonstrating their impact on the disease pathogenesis. Interestingly, diastolic parameters didn't show differences between male and female mice with HFpEF [62] or young and old female mice that received Ang II, but age did affect the LV response [61]. Estrogen status in females affects RAAS directly [59] and studies have demonstrated cardioprotective effects of estrogen in hypertension development [60]. It would be interesting to further study Ang II dose‐dependent alterations in the development of hypertensive heart disease in female mice.

Secondly, our study excluded the effect of metabolic factor, an important contributor to HFpEF development [9]. However, elimination of the metabolic effect gave us the opportunity to see a clear distinction between two developing phenotypes and to attribute cardiovascular pathophysiologies to Ang II dose or hypertrophy. Also, our study is limited to 4 weeks, which can't correlate with chronic HFpEF development in humans. During this period, we were able to achieve gradual blood pressure elevation, reaching levels of systemic hypertension similar to humans. Nevertheless, studies with longer Ang II exposure need to be conducted to disclose the further evolvement of the phenotypes.

## 5 Conclusion

In summary, this study underscores the critical role of hypertension in myocardial and arterial remodeling and in the development of hypertensive heart disease with progressing diastolic dysfunction and systemic HFpEF. Myocardial hypertrophy is a prominent mediator of disease progression in mice, together with hypertension, suggesting that the pathogenesis of HFpEF features also relies upon direct effects of Ang II. This indicates the potential of targeting Ang II for the treatment and risk prediction of concentric hypertrophy and diastolic dysfunction in both normotensive and hypertensive patients. Also, this study presents a feasible mouse model for studying cardiac and aortic modifications and their relation to parallelly progressing cardiac and extra‐cardiac pathologies—a subject not well studied in mice with hypertensive heart disease or HFpEF. In addition, the very limited amount of HFpEF‐specific treatment options and the inability of proposed therapies in clinical trials to reduce all‐cause or cardiovascular mortality [39, 63] emphasize the need to further investigate disease mechanisms and to develop mouse models for hypertensive HFpEF, especially those enabling the study of early disease pathology.

## Funding

This work was supported by the Research Council of Finland GeneCellNano Flagship Program (S.Y‐H.), Finnish Foundation for Cardiovascular Research (D.T., A‐K.R), Research Council of Finland (350049 to A‐K.R, 337120 to S.Y‐H.), Emil Aaltonen Foundation (to T.S.), Orion Research Foundation (to A‐K.R.), Aarne Koskelo Foundation (to D.T.), Päivikki and Sakari Sohlberg Foundation (to D.T.), and Aarne and Aili Turunen Foundation (to D.T.). This project has received funding from the European Union Horizon 2020 Framework Programme for Research and Innovation under grant agreement No 825670 (to S.Y‐H.).

## Ethics Statement

All animal experiments in the manuscript were approved by the National Experimental Animal Board of Finland and carried out following the guidelines of the Finnish Act on Animal Experimentation and Directive 2010/63/EU of the European Parliament under the license ESAVI‐2021‐014193.

## Conflicts of Interest

The authors declare no conflicts of interest.

## Supporting information


**Figure S1:** Systolic and diastolic blood pressure. Violin plot graphs for (A) systolic and (B) diastolic blood pressure measurements of individual mice (*n* = 5 for each group and time point).
**Figure S2:** Ang II did not affect plasma fasting glucose levels or liver enzymes. (A) Fasting blood glucose levels and (B) liver enzymes were measured in the end of the experiment in all groups.
**Figure S3:** Lung histopathology showed alveolar septal thickening in Ang II dose groups. (A) HE staining, magnification 40×, scale bar 100 μm. (B) Alveolar space was increased in HighA group C. Alveolar septal thickening was noticed in both dose groups. Data presented as mean ± SD (*n* = 7–11 mice/group; Statistical analysis was performed by One‐way ANOVA with Dunnett's post hoc test; quantification was performed from HE stained lung sections; for alveolar septal thickness, 5 measurements/mice were averaged; *p < 0.05, **p < 0.01).
**Table S1:** Body weight development within the groups. Data presented as mean ± SD (*n* = 7–10 mice/group; Statistical analysis was performed by paired two‐tailed Student's *t* test for comparison within the dose group and One‐way ANOVA with Dunnett's post hoc test for comparison between dose groups; **p < 0.01). *Comparison within the group.
**Table S2:** Heart weight and tibia length at the end of the experiment. Data presented as mean ± SD (*n* = 6–11 mice/group; Statistical analysis was performed by One‐way ANOVA with Dunnett's post hoc test for comparison between dose groups; **p < 0.01).
**Table S3:** Cardiac physiology data. Data presented as mean ± SD (*n* = 7–11 mice/group). Statistical analysis was performed by paired two‐tailed Student's *t* test for comparison within the dose group and One‐way ANOVA with Dunnett's post hoc test for comparison between dose groups *p < 0.05; **p < 0.01; ****p < 0.0001; *Comparison within the group. BP, during blood pressure measurement; BPM, beats per minute; d, in diastole; E, early mitral inflow velocity; e’, mitral annular early diastolic velocity; LVAW, left ventricular anterior wall; LVPW, left ventricular posterior wall; s, in systole.

## Data Availability

The data that support the findings of this study are available from the corresponding author upon reasonable request.

## References

[1] G. A. Roth , G. A. Mensah , C. O. Johnson , et al., “Global Burden of Cardiovascular Diseases and Risk Factors, 1990–2019: Update From the GBD 2019 Study,” Journal of the American College of Cardiology 76 (2020): 2982–3021. 33309175 10.1016/j.jacc.2020.11.010 PMC7755038

[2] F. D. Fuchs and P. K. Whelton , “High Blood Pressure and Cardiovascular Disease,” Hypertension 75 (2020): 285–292. 31865786 10.1161/HYPERTENSIONAHA.119.14240 PMC10243231

[3] A. Kasiakogias , E. A. Rosei , M. Camafort , et al., “Hypertension and Heart Failure With Preserved Ejection Fraction: Position Paper by the European Society of Hypertension,” Journal of Hypertension 39 (2021): 1522–1545. 34102660 10.1097/HJH.0000000000002910

[4] M. C. Tam , R. Lee , T. M. Cascino , M. C. Konerman , and S. L. Hummel , “Current Perspectives on Systemic Hypertension in Heart Failure With Preserved Ejection Fraction,” Current Hypertension Reports 19 (2017): 12. 28233237 10.1007/s11906-017-0709-2 PMC5503692

[5] P. L. Myhre , S. Selvaraj , and S. D. Solomon , “Management of Hypertension in Heart Failure With Preserved Ejection Fraction: Is There a Blood Pressure Goal?,” Current Opinion in Cardiology 36 (2021): 413–419. 33709982 10.1097/HCO.0000000000000852

[6] F. Triposkiadis , P. Sarafidis , A. Briasoulis , et al., “Hypertensive Heart Failure,” Journal of Clinical Medicine 12 (2023): 12. 10.3390/jcm12155090 PMC10419453 37568493

[7] A. B. Gevaert , J. R. A. Boen , V. F. Segers , and E. M. Van Craenenbroeck , “Heart Failure With Preserved Ejection Fraction: A Review of Cardiac and Noncardiac Pathophysiology,” Frontiers in Physiology 10 (2019): 10. 31191343 10.3389/fphys.2019.00638 PMC6548802

[8] I. Cuijpers , S. J. Simmonds , M. van Bilsen , et al., “Microvascular and Lymphatic Dysfunction in HFpEF and Its Associated Comorbidities,” Basic Research in Cardiology 115 (2020): 39. 32451732 10.1007/s00395-020-0798-y PMC7248044

[9] W. J. Paulus and M. R. Zile , “From Systemic Inflammation to Myocardial Fibrosis: The Heart Failure With Preserved Ejection Fraction Paradigm Revisited,” Circulation Research 128 (2021): 1451–1467. 33983831 10.1161/CIRCRESAHA.121.318159 PMC8351796

[10] D. Maeda , T. Dotare , Y. Matsue , et al., “Blood Pressure in Heart Failure Management and Prevention,” Hypertension Research 46 (2023): 817–833. 36604473 10.1038/s41440-022-01158-x

[11] C. Riehle and J. Bauersachs , “Small Animal Models of Heart Failure,” Cardiovascular Research 115 (2019): 1838–1849. 31243437 10.1093/cvr/cvz161 PMC6803815

[12] N. A. Noll , H. Lal , and W. D. Merryman , “Mouse Models of Heart Failure With Preserved or Reduced Ejection Fraction,” American Journal of Pathology 190 (2020): 1596–1608. 32343958 10.1016/j.ajpath.2020.04.006 PMC7416075

[13] M. Valero‐Muñoz , W. Backman , and F. Sam , “Murine Models of Heart Failure With Preserved Ejection Fraction A ‘Fishing Expedition’,” 6 (2017): 770–789. 10.1016/j.jacbts.2017.07.013 PMC5764178 29333506

[14] P. Ernsberger and R. J. Koletsky , “Metabolic Actions of Angiotensin Receptor Antagonists: PPAR‐γ Agonist Actions or a Class Effect?,” Current Opinion in Pharmacology 7 (2007): 140–145. 17303473 10.1016/j.coph.2006.11.008 PMC2930911

[15] S. Eguchi , T. Kawai , R. Scalia , and V. Rizzo , “Understanding Angiotensin II Type 1 Receptor Signaling in Vascular Pathophysiology,” Hypertension 71 (2018): 804–810. 29581215 10.1161/HYPERTENSIONAHA.118.10266 PMC5897153

[16] W. B. van Ham , E. L. Kessler , M. I. F. J. Oerlemans , et al., “Clinical Phenotypes of Heart Failure With Preserved Ejection Fraction to Select Preclinical Animal Models,” Basic to Translational Science 7 (2022): 844–857. 36061340 10.1016/j.jacbts.2021.12.009 PMC9436760

[17] Z. S. Galis , T. Thrasher , D. M. Reid , D. V. Stanley , and Y. S. Oh , “Investing in High Blood Pressure Research: A National Institutes of Health Perspective,” Hypertension 61 (2013): 757–761. 23438933 10.1161/HYPERTENSIONAHA.111.00770 PMC6711194

[18] G. Simon , G. Abraham , and G. Cserep , “Pressor and Subpressor Angiotensin II Administration Two Experimental Models of Hypertension,” American Journal of Hypertension 8 (1995): 645–650. 7662251 10.1016/0895-7061(95)00047-S

[19] D. J. Grieve , A. C. Cave , and A. M. Shah , “Cardiac Hypertrophy,” in A Handbook of Mouse Models of Cardiovascular Disease, ed. Q. Xu (Wiley, 2006).

[20] D. Weiss and R. W. Taylor , “Hypertension,” in A Handbook of Mouse Models of Cardiovascular Disease, ed. Q. Xu (Wiley, 2006).

[21] D. G. Harrison , M. Bader , L. O. Lerman , et al., “Tail‐Cuff Versus Radiotelemetry to Measure Blood Pressure in Mice and Rats,” Hypertension 81 (2024): 3–5. 37990918 10.1161/HYPERTENSIONAHA.123.22329 PMC10842069

[22] X. L. Merry Lindsey , Z. Kassiri , J. A. Virag , L. E. de Castro Brás , M. Scherrer‐Crosbie , and V. Jai , “Guidelines for Measuring Cardiac Physiology in Mice,” American Journal of Physiology. Heart and Circulatory Physiology 314 (2018): 733–752. 10.1152/ajpheart.00339.2017 PMC5966769 29351456

[23] R. M. Lang , L. P. Badano , M. A. Victor , et al., “Recommendations for Cardiac Chamber Quantification by Echocardiography in Adults: An Update From the American Society of Echocardiography and the European Association of Cardiovascular Imaging,” Journal of the American Society of Echocardiography 28 (2015): 1–39. 25559473 10.1016/j.echo.2014.10.003

[24] M. Nabati , S. Namazi , and J. Yazdani , “Aortic Wall Elasticity and Left Ventricular Function in Hypertensive Patients With Nonsignificant Coronary Artery Disease,” Ultrasound 29 (2021): 162–171. 34567228 10.1177/1742271X20963346 PMC8366218

[25] M. Merentie , J. A. Lipponen , M. Hedman , et al., “Mouse ECG Findings in Aging, With Conduction System Affecting Drugs and in Cardiac Pathologies: Development and Validation of ECG Analysis Algorithm in Mice,” Physiological Reports 3 (2015): 3. 10.14814/phy2.12639 PMC4760442 26660552

[26] J. Schindelin , I. Arganda‐Carreras , E. Frise , V. Kaynig , P. Tomancak , and A. Cardona , “Fiji: An Open‐Source Platform for Biological‐Image Analysis,” Nature Methods 9 (2012): 676–682. 22743772 10.1038/nmeth.2019 PMC3855844

[27] S. Vukelic and K. K. Griendling , “Angiotensin Ii, From Vasoconstrictor to Growth Factor: A Paradigm Shift,” Circulation Research 114 (2014): 754–757. 24577962 10.1161/CIRCRESAHA.114.303045 PMC3985550

[28] T. Kawai , S. J. Forrester , S. O'Brien , A. Baggett , V. Rizzo , and S. Eguchi , “AT1 Receptor Signaling Pathways in the Cardiovascular System,” Pharmacological Research 125 (2017): 4–13. 28527699 10.1016/j.phrs.2017.05.008 PMC5607088

[29] A. Tirronen , N. L. Downes , J. Huusko , et al., “The Ablation of Vegfr‐1 Signaling Promotes Pressure Overload‐Induced Cardiac Dysfunction and Sudden Death,” Biomolecules 11 (2021): 1–15. 10.3390/biom11030452 PMC8002705 33802976

[30] T. Sumida , A. T. Naito , S. Nomura , A. Nakagawa , T. Higo , and I. Komuro , “Complement C1q‐Induced Activation of β‐Catenin Signalling Causes Hypertensive Arterial Remodelling,” Nature Communications 6 (2015): 6. 10.1038/ncomms7241 PMC4351572 25716000

[31] P. Moritz Becher , D. Lindner , K. Miteva , et al., “Role of Heart Rate Reduction in the Prevention of Experimental Heart Failure‐Comparison Between I f‐Channel Blockade And‐Receptor,” Hypertension 59 (2012): 949–957. 22493071 10.1161/HYPERTENSIONAHA.111.183913

[32] D. Westermann , P. M. Becher , D. Lindner , et al., “Selective PDE5A Inhibition With Sildenafil Rescues Left Ventricular Dysfunction, Inflammatory Immune Response and Cardiac Remodeling in Angiotensin II‐Induced Heart Failure In Vivo,” Basic Research in Cardiology 107 (2012): 308. 23117837 10.1007/s00395-012-0308-y

[33] M. Van Bilsen , A. Daniels , O. Brouwers , and B. Janssen , “Hypertension Is a Conditional Factor for the Development of Cardiac Hypertrophy in Type 2 Diabetic Mice,” PLoS One 9 (2014): e85078. 24416343 10.1371/journal.pone.0085078 PMC3887022

[34] M. Mishra , I. Muthuramu , H. Kempen , and B. De Geest , “Administration of Apo A‐I (Milano) Nanoparticles Reverses Pathological Remodelling, Cardiac Dysfunction, and Heart Failure in a Murine Model of HFpEF Associated With Hypertension,” Scientific Reports 10 (2020): 10. 32433476 10.1038/s41598-020-65255-y PMC7239951

[35] F. Olivares‐Silva , N. De Gregorio , J. Espitia‐Corredor , et al., “Resolvin‐D1 Attenuation of Angiotensin II‐Induced Cardiac Inflammation in Mice Is Associated With Prevention of Cardiac Remodeling and Hypertension,” Biochimica et Biophysica Acta, Molecular Basis of Disease 8 (2021): 1867. 10.1016/j.bbadis.2021.166241 34400298

[36] J. Regan , A. Gabriele Mauro , S. Carbone , et al., “A Mouse Model of Heart Failure With Preserved Ejection Fraction due to Chronic Infusion of a Low Subpressor Dose of Angiotensin II,” American Journal of Physiology. Heart and Circulatory Physiology 309 (2015): H771–H778. 26188021 10.1152/ajpheart.00282.2015 PMC4591411

[37] A. M. Shah , M. Cikes , N. Prasad , G. Li , S. Getchevski , and B. Claggett , “Echocardiographic Features of Patients With Heart Failure and Preserved Left Ventricular Ejection Fraction,” Journal of the American College of Cardiology 74 (2019): 2858–2873. 31806129 10.1016/j.jacc.2019.09.063 PMC13537700

[38] C. Lam , V. Roger , R. Rodeheffer , F. Bursi , “Cardiac Structure and Ventricular‐Vascular Function in Persons With Heart Failure and Preserved Ejection Fraction From Olmsted County, Minnesota,” Circulation 115 (2007): 1982–1990. 17404159 10.1161/CIRCULATIONAHA.106.659763 PMC2001291

[39] M. Redfield and B. Borlaug , “Heart Failure With Preserved Ejection Fraction: A Review,” Journal of the American Medical Association 3 (2023): 827–838. 10.1001/jama.2023.2020 36917048

[40] M. Zile , C. Baicu , J. Ikonomidis , et al., “Myocardial Stiffness in Patients With Heart Failure and a Preserved Ejection Fraction Contributions of Collagen and Titin,” Circulation 131 (2015): 1247–1259. 25637629 10.1161/CIRCULATIONAHA.114.013215 PMC4390480

[41] M. Eppenberger‐Eberhardt , I. Flamme , V. Kurer , and H. M. Eppenberger , “Reexpression of α‐Smooth Muscle Actin Isoform in Cultured Adult Rat Cardiomyocytes,” Developmental Biology 139 (1990): 269–278. 2186943 10.1016/0012-1606(90)90296-u

[42] F. Black , S. Packer , and T. Parker , “The Vascular Smooth Muscle α‐Actin Gene Is Reactivated During Cardiac Hypertrophy Provoked by Load,” Journal of Clinical Investigation 88 (1991): 1581–1588. 1834699 10.1172/JCI115470 PMC295677

[43] A. Suurmeijer , S. Clément , A. Francesconi , et al., “α‐Actin Isoform Distribution in Normal and Failing Human Heart: A Morphological, Morphometric, and Biochemical Study,” Journal of Pathology 199 (2003): 387–397. 12579541 10.1002/path.1311

[44] S. H. Wan , M. W. Vogel , and H. H. Chen , “Pre‐Clinical Diastolic Dysfunction,” Journal of the American College of Cardiology 63 (2014): 407–416. 24291270 10.1016/j.jacc.2013.10.063 PMC3934927

[45] T. McDonagh , M. Metra , M. Adamo , et al., “2021 ESC Guidelines for the Diagnosis and Treatment of Acute and Chronic Heart Failure,” European Heart Journal 42 (2021): 3599–3726. 34447992 10.1093/eurheartj/ehab368

[46] M. Schnelle , N. Catibog , M. Zhang , et al., “Echocardiographic Evaluation of Diastolic Function in Mouse Models of Heart Disease,” Journal of Molecular and Cellular Cardiology 114 (2018): 20–28. 29055654 10.1016/j.yjmcc.2017.10.006 PMC5807035

[47] M. Villalba‐Orero , P. Garcia‐Pavia , and E. Lara‐Pezzi , “Non‐Invasive Assessment of HFpEF in Mouse Models: Current Gaps and Future Directions,” BMC Medicine 20 (2022): 349. 36229816 10.1186/s12916-022-02546-3 PMC9563110

[48] L. Zhihao , N. Jingyu , L. Lan , et al., “SERCA2a: A Key Protein in the Ca2+ Cycle of the Heart Failure,” Heart Failure Reviews 25 (2020): 523–535. 31701344 10.1007/s10741-019-09873-3

[49] L. Stoicescu , D. Crişan , C. Morgovan , L. Avram , and S. Ghibu , “Heart Failure With Preserved Ejection Fraction: The Pathophysiological Mechanisms Behind the Clinical Phenotypes and the Therapeutic Approach,” International Journal of Molecular Sciences 25 (2024): 25. 10.3390/ijms25020794 PMC10815792 38255869

[50] X. Zuo , L. Liu , K. Liu , et al., “Proximal Aorta Dilatation in Hypertension,” Journal of Hypertension 41 (2023): 1511–1520. 37642588 10.1097/HJH.0000000000003518

[51] I. Karagodin , O. Aba‐Omer , R. Sparapani , and J. L. Strande , “Aortic Stiffening Precedes Onset of Heart Failure With Preserved Ejection Fraction in Patients With Asymptomatic Diastolic Dysfunction,” BMC Cardiovascular Disorders 17 (2017): 62. 28196483 10.1186/s12872-017-0490-9 PMC5310057

[52] A. Schulz , I. Schellinger , and S. Backhaus , “Association of Cardiac MRI–Derived Aortic Stiffness With Early Stages and Progression of Heart Failure With Preserved Ejection Fraction,” Radiology: Cardiothoracic Imaging 6 (2024): 6. 10.1148/ryct.230344 PMC11369653 39145733

[53] T. He , C. Liu , and W. Liang , “Abnormal Electrocardiogram and Poor Prognosis in Heart Failure With Preserved Ejection Fraction,” Postgraduate Medical Journal 99 (2023): 1154–1159. 37427981 10.1093/postmj/qgad055

[54] J. Díez , A. González , and J. C. Kovacic , “Myocardial Interstitial Fibrosis in Nonischemic Heart Disease, Part 3/4: JACC Focus Seminar,” Journal of the American College of Cardiology 75 (2020): 2204–2218. 32354386 10.1016/j.jacc.2020.03.019 PMC7213023

[55] S. Verheule and U. Schotten , “Electrophysiological Consequences of Cardiac Fibrosis,” Cells 10 (2021): 10. 10.3390/cells10113220 PMC8625398 34831442

[56] J. C. B. Jacobsen , I. H. Schubert , K. Larsen , D. Terzic , L. Thisted , and M. B. Thomsen , “Preload Dependence in an Animal Model of Mild Heart Failure With Preserved Ejection Fraction (HFpEF),” Acta Physiologica 240 (2024): e14099. 38230889 10.1111/apha.14099

[57] G. Zhan , X. Wang , X. Wang , et al., “Dapagliflozin: A Sodium–Glucose Cotransporter 2 Inhibitor, Attenuates Angiotensin II‐Induced Atrial Fibrillation by Regulating Atrial Electrical and Structural Remodeling,” European Journal of Pharmacology 978 (2024): 176712. 38906237 10.1016/j.ejphar.2024.176712

[58] J. I. Emerson , P. Ariel , W. Shi , and F. L. Conlon , “Sex Differences in Mouse Cardiac Electrophysiology Revealed by Simultaneous Imaging of Excitation‐Contraction Coupling,” Journal of Cardiovascular Development and Disease 10 (2023): 10. 10.3390/jcdd10120479 PMC10743987 38132647

[59] P. J. Connelly , G. Currie , and C. Delles , “Sex Differences in the Prevalence, Outcomes and Management of Hypertension,” Current Hypertension Reports 24 (2022): 185–192. 35254589 10.1007/s11906-022-01183-8 PMC9239955

[60] B. Xue , A. K. Johnson , M. Hay , and A. K. Johnson , “Sex Differences in Angiotensin II‐ and Aldosterone‐Induced Hypertension: The Central Protective Effects of Estrogen,” American Journal of Physiology. Regulatory, Integrative and Comparative Physiology 305 (2013): 459–463. 10.1152/ajpregu.00222.2013 PMC3763030 23883676

[61] É. Walsh‐Wilkinson , M. Lamine Aidara , A. Morin‐Grandmont , et al., “Age and Sex Hormones Modulate Left Ventricle Regional Response to Angiotensin II in Male and Female Mice,” American Journal of Physiology. Heart and Circulatory Physiology 323 (2022): H643–H658. 35984762 10.1152/ajpheart.00044.2022

[62] D. Matsiukevich , A. Kovacs , T. Li , et al., “Characterization of a Robust Mouse Model of Heart Failure With Preserved Ejection Fraction,” American Journal of Physiology. Heart and Circulatory Physiology 325 (2023): H203–H231. 37204871 10.1152/ajpheart.00038.2023 PMC11932539

[63] A. B. Gevaert , R. Kataria , F. Zannad , et al., “Heart Failure With Preserved Ejection Fraction: Recent Concepts in Diagnosis, Mechanisms and Management,” Heart 108 (2022): 1342–1350. 35022210 10.1136/heartjnl-2021-319605

