# Supplementary material for: Low and High Pressor Doses of Ang II Lead to Two Distinct Phenotypes of Hypertensive Heart Disease in Mice
Source: APMIS. 2026 Jan 6;134(1):e70132. doi: 10.1111/apm.70132 (PMC12774864; doi:10.1111/apm.70132)
Supplement: Supplementary file 1 — Figure S1: Systolic and diastolic blood pressure. Violin plot graphs for (A) systolic and (B) diastolic blood pressure measurements of individual mice (n = 5 for each group and time point). Figure S2: Ang II did not affect plasma fasting glucose levels or liver enzymes. (A) Fasting blood glucose levels and (B) liver enzymes were measured in the end of the experiment in all groups. Figure S3: Lung histopathology showed alveolar septal thickening in Ang II dose groups. (A) HE staining, magnification 40×, scale bar 100 μm. (B) Alveolar space was increased in HighA group C. Alveolar septal thickening was noticed in both dose groups. Data presented as mean ± SD (n = 7–11 mice/group; Statistical analysis was performed by One‐way ANOVA with Dunnett's post hoc test; quantification was performed from HE stained lung sections; for alveolar septal thickness, 5 measurements/mice were averaged; *p < 0.05, **p < 0.01). Table S1: Body weight development within the groups. Data presented as mean ± SD (n = 7–10 mice/group; Statistical analysis was performed by paired two‐tailed Student's t test for comparison within the dose group and One‐way ANOVA with Dunnett's post hoc test for comparison between dose groups; **p < 0.01). *Comparison within the group. Table S2: Heart weight and tibia length at the end of the experiment. Data presented as mean ± SD (n = 6–11 mice/group; Statistical analysis was performed by One‐way ANOVA with Dunnett's post hoc test for comparison between dose groups; **p < 0.01). Table S3: Cardiac physiology data. Data presented as mean ± SD (n = 7–11 mice/group). Statistical analysis was performed by paired two‐tailed Student's t test for comparison within the dose group and One‐way ANOVA with Dunnett's post hoc test for comparison between dose groups *p < 0.05; **p < 0.01; ****p < 0.0001; *Comparison within the group. BP, during blood pressure measurement; BPM, beats per minute; d, in diastole; E, early mitral inflow velocity; e’, mitral annular early diastolic [file APM-134-0-s001.docx]

**SUPPLEMENTAL INFORMATION**

**Titles**

Figure S1

Figure S2

Figure S3

Table S1

Table S2

Table S3

**Figure S1.**

**A.
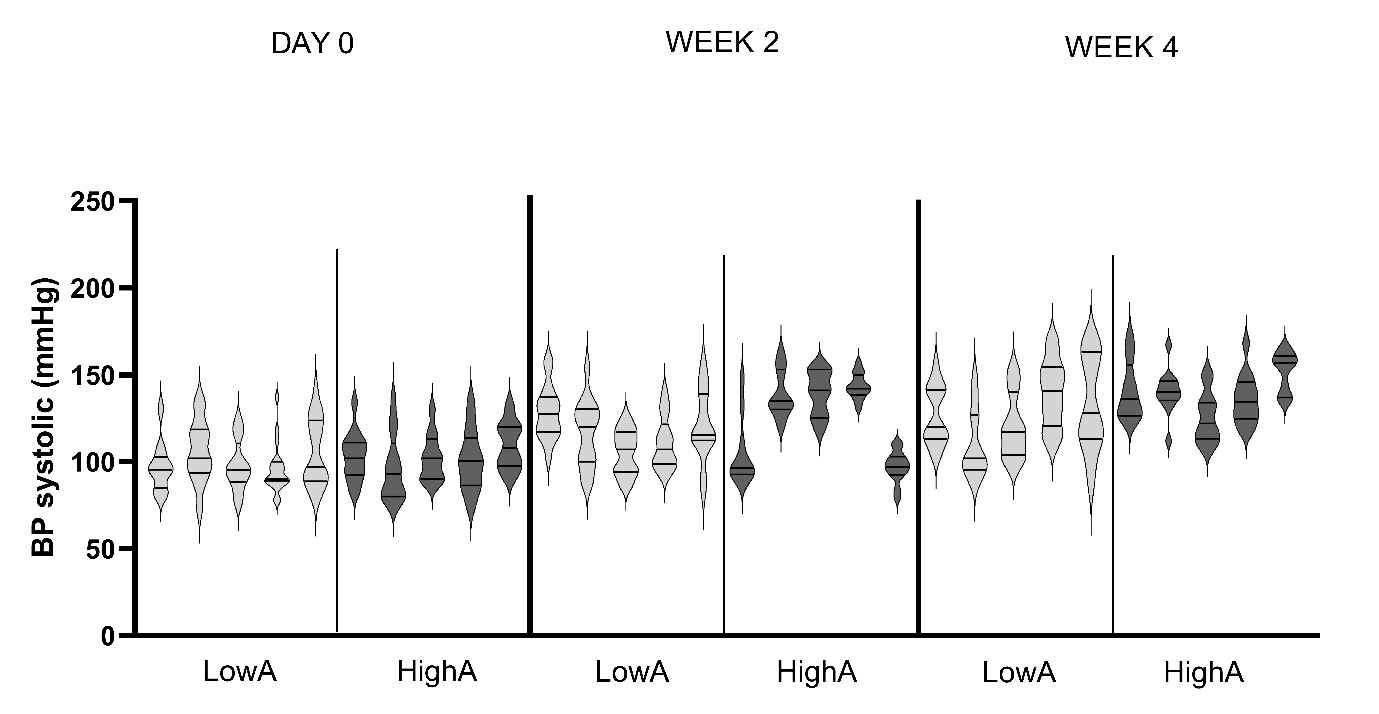
**

**B.**

**
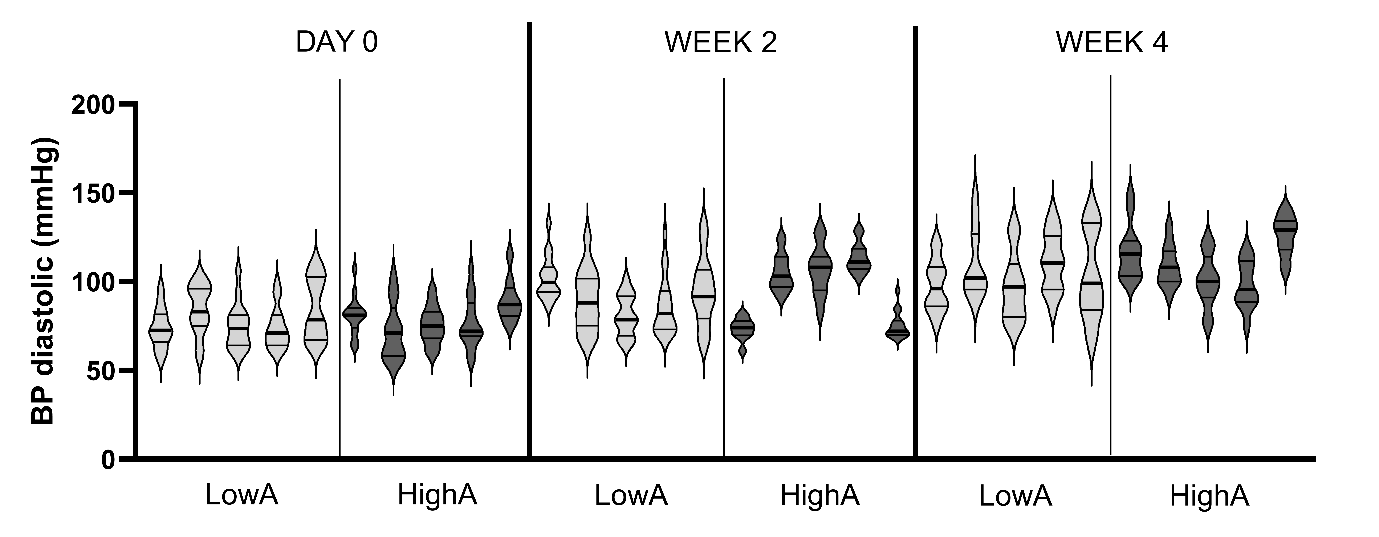
**

**Figure S2.**

**
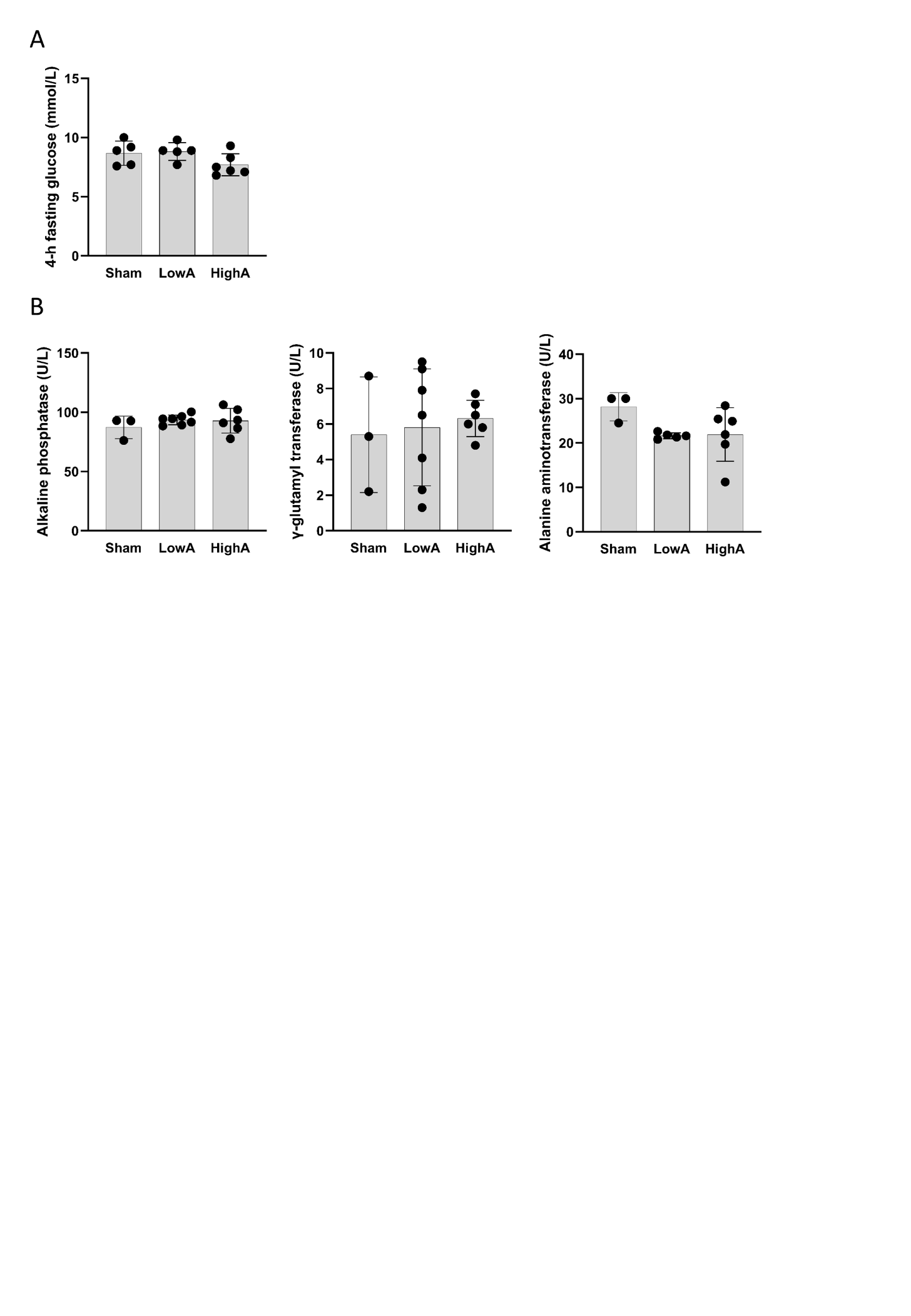
**

**S3.**

**A.**


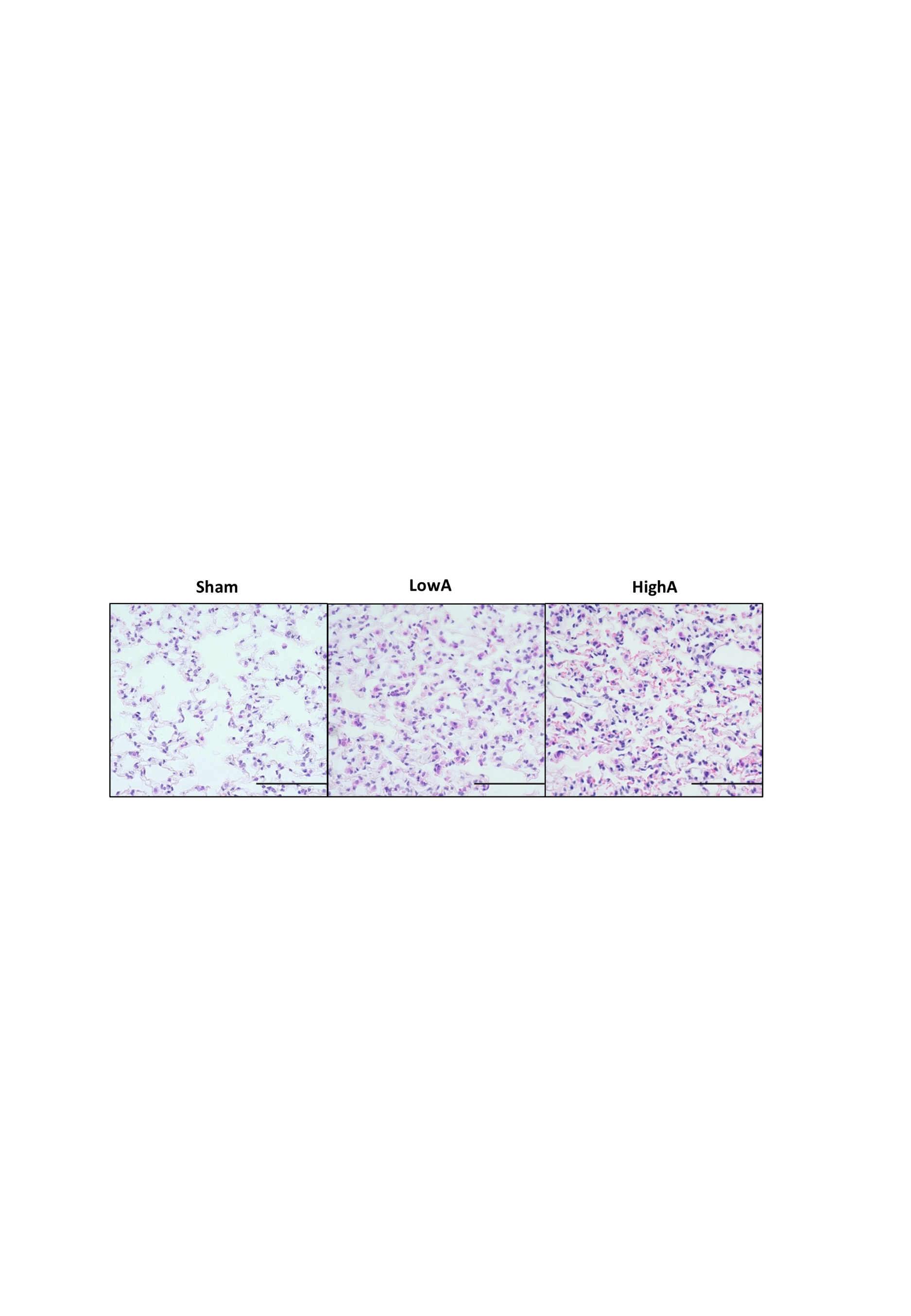


*100 µm*

**
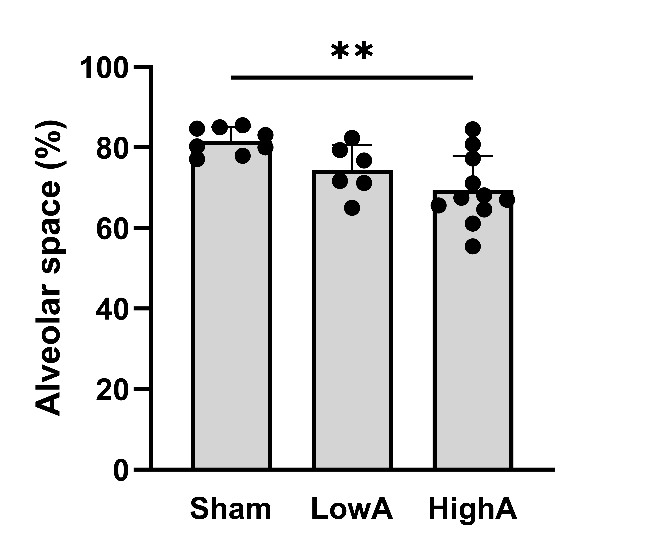

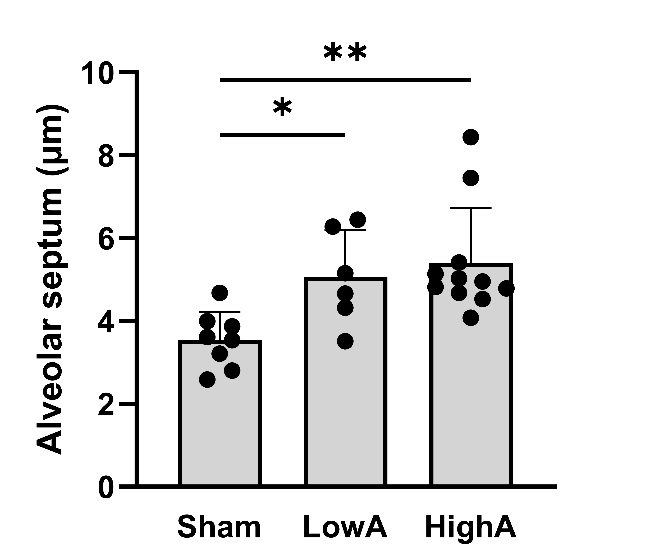
B. C.**

**SUPPLEMENTAL INFORMATION**

**Legends**

**Figure S1. Systolic and diastolic blood pressure.** Violin plot graphs for **A:** systolic and **B:** diastolic blood pressure measurements of individual mice (n=5 for each group and time point)

**Figure S2. Ang II did not affect plasma fasting glucose levels or liver enzymes.** **A:** Fasting blood glucose levels and **B:** liver enzymes were measured in the end of the experiment in all groups.

**Figure S3. Lung histopathology showed alveolar septal thickening in Ang II dose groups. A:** HE staining, magnification 40x, scale bar 100 $\mu$m. **B.** Alveolar space was increased in HighA group **C.** Alveolar septal thickening was noticed in both dose groups. Data presented as mean ± SD (n= 7-11 mice/group; Statistical analysis was performed by One-way ANOVA with Dunnett’s post-hoc test; quantification was performed from HE stained lung sections; for alveolar septal thickness, 5 measurements/mice were averaged; **p<0.05, **p<0.01*).

| **Mean ± SD** | **Day 0 (g)** | **Week 1 (g)** | **Week 2 (g)** | **Week 3 (g)** | **Week 4 (g)** |
| --- | --- | --- | --- | --- | --- |
| LowA | 28.3 ± 1.8 | 28.8 ± 1.7 | 29.4 ± 2.3 | 29.7 ± 2.4 | 29.1 ± 2.3 |
| HighA | 28.3 ± 1.2 | 28.1 ± 0.8 | 28.6 ± 1.1 | 27.7 ± 1.4 | 25.9 ± 1.9 ** |

**Table S1. Body weight development within the groups.** Data presented as mean ± SD (n = 7-10 mice/group; Statistical analysis was performed by paired two-tailed Student’s *t* test for comparison within the dose group and One-way ANOVA with Dunnett’s post-hoc test for comparison between dose groups; ***p<0.01*). * comparison within the group.

| **Mean ± SD** | Heart weight (mg) | Tibia length (mm) |
| --- | --- | --- |
| Sham | 157.20 ± 16.17 | 20 ± 0 |
| LowA | 183.20 ± 29.90 | 20 ± 0 |
| HighA | 205.50 ± 34.61 ** | 20 ± 0 |

**Table S2. Heart weight and tibia length at the end of the experiment.** Data presented as mean ± SD (n = 6-11 mice/group; Statistical analysis was performed by One-way ANOVA with Dunnett’s post-hoc test for comparison between dose groups; ***p<0.01*).

|  | **Group** | **LowA** | | **HighA** | |
| --- | --- | --- | --- | --- | --- |
| **Parameter** | **Unit** | **Day 0** | **Week 4** | **Day 0** | **Week 4** |
| Heart rate | *BPM* | 399 ± 55 | 455 ± 42 | 386 ± 58 | 438 ± 71 |
| Heart rate (BP) | *BPM* | 445 ± 95 | 445 ± 114 | 491 ± 84 | 396 ± 73 |
| Diameter, s | *mm* | 3.00 ± 0.28 | 2.72 ± 0.20 | 2.85 ± 0.42 | 2.48 ± 0.43 |
| Volume, s | *μL* | 35.5 ± 7.8 | 27.7 ± 4.8 | 32.0 ± 11.3 | 23.1 ±10.4 |
| LVAW, s | *mm* | 1.37 ± 0.10 | 1.53 ± 0.17 | 1.40 ± 0.22 | 1.61 ± 0.27* |
| LVAW, d | *mm* | 0.95 ± 0.09 | 0.99 ± 0.19 | 1.01 ± 0.19 | 1.16 ± 0.24* |
| LVPW, s | *mm* | 1.26 ± 0.13 | 1.41 ± 0.29 | 1.16 ± 0.12 | 1.70 ± 0.26 **** |
| E | *mm/s* | 656 ± 80 | 656 ± 92 | 689 ± 95 | 559 ± 148* |
| e’ | *mm/s* | 20.1 ± 6.0 | 20.7 ± 4.0 | 22.6 ± 7.7 | 15.6 ± 5.6** |

**Table S3. Cardiac physiology data.** Data presented as mean ± SD (n=7-11 mice/group). Statistical analysis was performed by paired two-tailed Student’s *t* test for comparison within the dose group and One-way ANOVA with Dunnett’s post-hoc test for comparison between dose groups **p<0.05; **p<0.01; ****p<0.0001*); * comparison within the group. LVAW, left ventricular anterior wall; LVPW, left ventricular posterior wall; s, in systole; d, in diastole; E, early mitral inflow velocity; e’, mitral annular early diastolic velocity; BP, during blood pressure measurement; BPM, beats per minute.
